# Photoinduced Electron Transfer Informs on Pathway Coupling in Flavin-Based Electron Bifurcation

**DOI:** 10.1021/acsbiomedchemau.5c00232

**Published:** 2026-02-06

**Authors:** Seth A. Wiley, Carolyn E. Lubner

**Affiliations:** Biosciences Center, 53405 National Laboratory of the Rockies, Golden, Colorado 80401, United States

**Keywords:** Electron Bifurcation, Redox Reactions, Reaction Intermediates, Photochemistry, Mechanism, Proteins, Enzymes

## Abstract

Flavin-based electron bifurcation (FBEB) is an enzymatic mechanism that generates extremely high-energy electrons to drive unfavorable chemical reactions. It is utilized by the NADH-dependent ferredoxin:NADP^+^-oxidoreductase (Nfn) enzyme in hyperthermophile *Pyrococcus furiosus* to bifurcate electrons from NADPH into the coupled low-potential (endergonic) and high-potential (exergonic) pathways. This process enables *P. furiosus* to live in harsh and uninviting environments. Despite its biological importance, the mechanisms used by Nfn to facilitate exceptional directional control over short-lived, high-energy electrons and to prevent undesired transfer, particularly along the low-potential pathway, are still not well understood. To elucidate how the protein environment contributes to electronic control in the low-potential pathway, new techniques must be utilized to probe these unstable intermediates. In this study, we have adapted low-temperature photoexcitation combined with electron paramagnetic resonance (EPR) to accumulate the short-lived intermediate and place it in the context of the other cofactors involved in the low-potential pathway of Nfn. We observed coincident growth of both the radical intermediate and its nearby [4Fe-4S] cluster over 4.5 h of illumination with NADPH at cryogenic temperatures. The photogenerated paramagnetic species were stable in LN_2_ storage indefinitely and recombined when warmed to higher temperatures. The results provide insights into the electron transfer steps and cofactor interactions along the low potential pathway, facilitating a more robust mechanistic understanding of the high-energy events of electron bifurcation. Furthermore, through comparison of cryogenic and room temperature experiments, a potential gating step involving the movement of key residues important for the reversibility of electron flow along this pathway is suggested.

## Graphical abstract

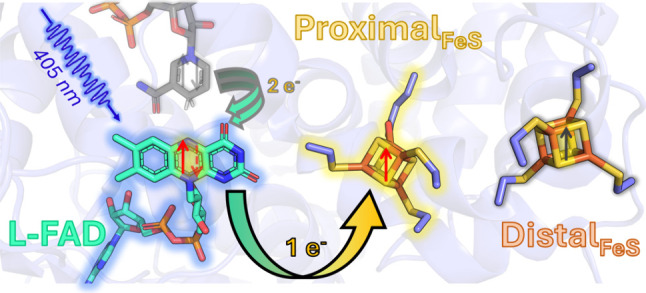

## Introduction

The ability to control the direction of energy flow is a crucial part of how organisms maintain homeostasis and adapt to dynamic or extreme environments, such as hydrothermal vents.^1,2^ Critically, to survive in such conditions, organisms must control the energetic flux to gain every advantage they can while preventing unnecessary energy loss. Over the past 50+ years^3,4^ there has been much interest in determining how these energy-efficient biochemical processes occur, and more recently, a lot of attention has been focused on the phenomenon of electron bifurcation.^1,5^

Electron bifurcation is a mechanism of energy conservation many organisms use to minimize energy loss during interconversion of cellular currencies for metabolic processes.^1^ Bifurcation promotes the reversible conversion of a middle-energy starting material/substrate to a coupled pair of high- and low-energy reducing equivalents that the cell can use for more energy-demanding processes.^2^ In this mechanism, the overall thermodynamic landscape dictates how the energetic sacrifice in one electron is coupled to the increase of energy in the other, resulting in an energetic redistribution between the electrons.^6,7^ At the thermodynamic limits of life, many microbes exploit electron bifurcation to redistribute energetic equivalents and increase the overall efficiency of their metabolic processes.^5^ Flavin-Based Electron Bifurcation (FBEB) uses a flavin cofactor as the site of electron bifurcation^8^ which are apt for facilitating both 1- and 2-electron transfers by way of their 3 predominant redox states: oxidized quinone (OX), 1-electron reduced semiquinone (SQ), and 2-electron reduced hydroquinone (HQ).^9,10^

In the hyperthermophillic organism *Pyrococcus furiosus*, FBEB is performed by the NADH-dependent ferredoxin:NADP^+^-oxidoreductase complex I, also known as NfnSL (Figure 1).^11^ This dimeric enzyme contains two [4Fe-4S] clusters in the large subunit, one [2Fe-2S] cluster in the small subunit, and two flavin adenine dinucleotides (FAD), one in each subunit (Figure 1A), assigned respectively as the L-FAD in NfnL and the S-FAD in NfnS. Overall, the NfnSL complex carries out NADPH oxidation coupled to the simultaneous reduction of NAD^+^ and ferredoxin (Fd) (eq 1; Figure 1C).

1 $$2NADPH+NAD^{+}+2Fd_{ox}↔2NADP^{+}+NADH+H^{+}+2Fd_{red}$$

**Figure 1:**
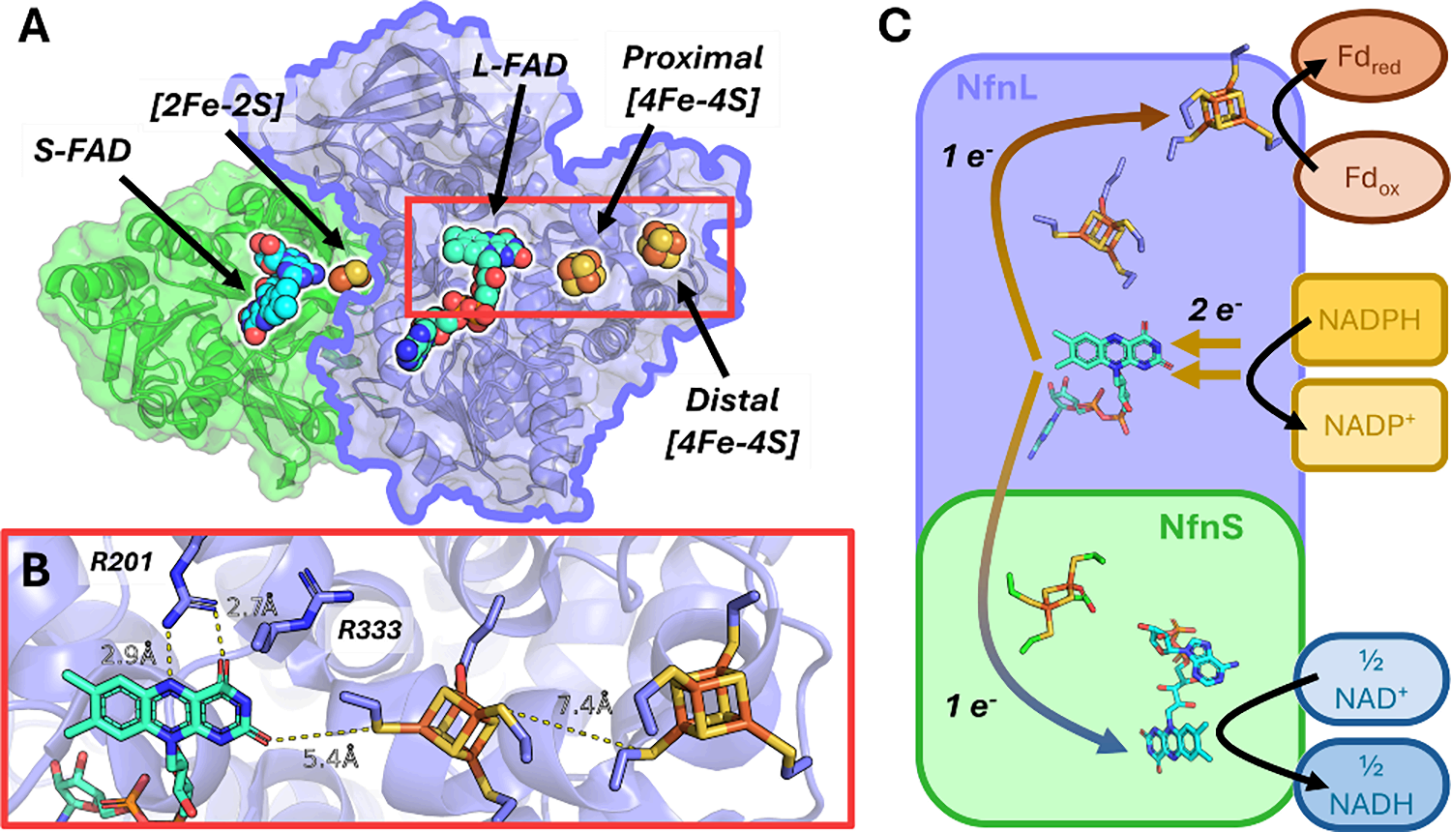
NfnSL from *Pyrococcus furiosus*. Panel **A** shows the NfnSL complex containing NfnS (green) and NfnL (blue), with NfnS containing the [2Fe-2S] cluster and S-FAD, and NfnL containing the 2x [4Fe-4S] clusters and L-FAD, respectively. The low-potential pathway is located in the NfnL subunit, with the pathway and key residues, R201 and R333, and key distances highlighted in panel **B**. The 5.4 Å distance was measured between the O4' to C101 sulfur (7.4 Å O4' to Fe). Panel **C** depicts the overall reaction catalyzed by the NfnSL complex, with the L-FAD as the site of electron bifurcation (center), the high-potential pathway on the bottom in NfnS (blue arrows), and the low-potential pathway on top in NfnL (rust arrows). Once the L-FAD is reduced, one electron transfers “downhill” to the [2Fe-2S] cluster in NfnS, and then the other electron transfers from the L-FAD to the nearby proximal [4Fe-4S] cluster, then to the distal [4Fe-4S] cluster, and ultimately to a partner ferredoxin. NfnL is outlined in panel **A** as it is the only subunit used in the experiments in this report. PDB:5JFC.

The subunits of the NfnSL complex are effectively separated into the high-potential pathway in NfnS and the low-potential pathway in NfnL, with the bifurcating flavin, L-FAD, located in NfnL at the fork between the two pathways. The bifurcation reaction is triggered by a spontaneous downhill electron transfer (ET) through the high-potential pathway, leaving a transient, unstable anionic semiquinone (ASQ) radical on the L-FAD, which then rapidly undergoes ET via the low-potential pathway to reduce externally located Fd.^11,12^ Recently, much has been learned about the thermodynamic landscape in NfnSL, particularly regarding how the cofactors are poised for electron transfer.^11^ The midpoint redox potentials of the high- and low-potential pathways in NfnSL have been investigated by square-wave voltammetry, showing that within the low-potential pathway the iron–sulfur cluster closest to the L-FAD, the differentially ligated proximal [4Fe-4S] cluster, has a midpoint potential of ∼ −700 mV, and that the cluster further away, the distal [4Fe-4S] cluster, has a midpoint potential of ∼ −520 mV.^13^ The L-FAD has been found to have crossed-potentials, where under equilibrium conditions only the oxidized (“L-FAD_OX_”) and hydroquinone states (“L-FAD_HQ_”), but not the semiquinone state (“L-FAD_SQ_”), are observed, with a 2 e^–^ midpoint redox potential of −435 mV.^13^ Despite the L-FAD showing only 2 electron chemistry under equilibrium conditions, ultrafast transient absorption spectroscopy (TAS) has shown the existence of a highly unstable ASQ with a lifetime of only 10 ps, that was coincident with an L-FAD_OX_ bleach.^11,13,14^ Based on the 7.5 Å edge-to-edge distance between the L-FAD and the proximal [4Fe-4S] cluster, the proximal [4Fe-4S] cluster’s midpoint potential, and the lifetime (10 ps) of the ASQ, the L-FAD OX to ASQ midpoint potential of −911 mV was calculated using the Moser-Dutton equation.^11,13^ These recent findings have revealed the low-potential pathway in NfnL is poised for rapid, downstream electron transfer once the electron pair is separated and the ASQ is generated.^13^

This rapid electron transfer in the low potential pathway is further controlled by key residues located within the cofactor microenvironments, such as the site-differentiation of the proximal [4Fe-4S] cluster^15^ and the residues surrounding the L-FAD.^11^ The role of site-differentiation in pathway coupling has been investigated, with key studies showing how the high-potential pathway^12^ and the site-differentiated ligand on the proximal [4Fe-4S] cluster^15,16^ dictate the control of electron transfer in NfnSL. Without the high-potential electron acceptor, NAD^+^, NfnSL is capable of only 3 bifurcation events before stalling due to the full occupancy of the high-potential pathway cofactors (1x [2Fe-2S] and 2x S-FAD).^12^ When the proximal [4Fe-4S] cluster’s unique glutamic acid ligation is substituted to the canonical cysteine, the low- and high-potential pathways appear to decouple, leading to bifurcation activity without the high-potential electron acceptor.^16^ How NfnSL tunes the environments surrounding the redox cofactors to control electron transfer, in particular, the initial high-energy steps of electron bifurcation, is critical for exploiting and expanding this unique chemical transformation mechanism.

Two notable arginine residues, R201 and R333 (Figure 1B), either directly interface with L-FAD N5 and O4’ (R201)^11^ or are highly conserved *only* in electron bifurcating enzymes (R333)^17^ and appear to be critical for facilitating, tuning, and stabilizing the transient ASQ intermediate on the L-FAD. How nearby arginines and the [4Fe-4S] site-differentiated glutamate residue influence electronic control and directional bias are of significant interest, but to gain insight into each residue’s contribution to the overall mechanism, methods to interrogate the high-energy electron transfer steps must be realized.

Ultrafast tools, like femtosecond laser spectroscopy, have proved indispensable for probing transient intermediates and enzyme dynamics of light-activated enzymes with high temporal resolution.^18^ In addition, these methods can elucidate the sequence of events following photon absorption, providing the critical temporal context to the steps underlying enzymatic mechanisms postactivation.^18^ While ultrafast TAS experimental evidence allowed for determination of the mechanistically relevant L-FAD intermediate, the short lifetime has complicated the assessment and investigation of the impact of the surrounding redox environment on enabling this ASQ for subsequent electron transfer down the low-potential pathway. How these nearby residues and cofactors within the low-potential pathway interact with the ASQ intermediate and prevent short-circuit electron transfer to the high-potential pathway is currently unknown. To better understand how energy is coupled in electron bifurcating systems, it is necessary to study the high energy electron transfer step from the ASQ to the proximal [4Fe-4S] cluster. As photoexcitation has been previously used to study many photosensitive biomolecules and proteins,^19–21^ we aimed to adapt cryo-photoexcitation methods^21–23^ to an FBEB enzyme and utilize the intrinsic photophysical properties of the L-FAD and cryogenic temperatures to trap and study the mechanistically relevant intermediates by electron paramagnetic resonance (EPR) spectroscopy. EPR spectroscopy allows for monitoring of characteristic spectral features (known as *g*-values) for a variety of paramagnetic species, such as the [4Fe-4S] clusters and flavin radical species. Tracking these paramagnetic species sheds new light on the mechanism of bifurcation, the low-potential pathway’s electron transfer network, and allows for determining fundamental thermodynamic, physical, and electronic characteristics of key bifurcation intermediates.

In this report, we have observed reversible photoinduced electron transfer, showing the unambiguous generation of both the ASQ and proximal [4Fe-4S] cluster signals in the isolated NfnL subunit in the presence of NADPH. The signals for these photogenerated species increased in intensity with illumination time, and when stored at liquid nitrogen temperatures (∼77 K), the ASQ and proximal cluster species remained stable without losing intensity. Annealing led to pronounced recombination within the low-potential pathway, signifying a physical gating step in the directionality of electron transfer. The outcomes gained from this work expand the model of pathway coupling and electron transfer control in FBEB.

## Results

To investigate the low-potential pathway and unambiguously probe the bifurcating L-FAD without interference from the additional S-FAD in NfnS, we performed studies utilizing only the NfnL subunit. We have previously shown that this methodology results in full incorporation of the NfnL cofactors, and the binding with NADP­(H), L-FAD intermediate identity (ASQ) and lifetime, and cofactor reduction potentials are unaltered compared to NfnSL.^13,16^

### L-FAD ASQ and Proximal [4Fe-4S] Cluster Signals Form upon Photoexcitation

Previous experiments detected reduction of only the distal [4Fe-4S] cluster in the presence of NADPH.^13^ To probe the transient ASQ observed via ultrafast TAS experiments,^11,13^ we photoexcited the oxidized L-FAD present in NfnL in the presence of its native substrate, NADPH. These experiments were conducted at 233 K to separate electron transfer processes from structural changes to have the best chance of trapping high energy intermediates.^22^ As depicted in Figure 2, upon photoexcitation of NfnL in the presence of 8.8 mM NADPH at 233 K with 2 W 405 nm illumination for 1 h, two new species arose on top of the existing rhombic distal [4Fe-4S] cluster signal with *g*-values of 2.034, 1.934, and 1.896 (Figures S1 and S2, Table S2): one isotropic, organic radical-like spectrum with a *g*_iso_ = 2.0033, and another resembling the previously observed proximal [4Fe-4S] cluster with newly reassigned semiaxial *g*-values corresponding to 1.972, 1.954, and 1.924 (Figures S3–S6, Table S3). These two emergent signals were distinct, and each of the two species had unique relaxation characteristics consistent with an organic radical, assigned to the L-FAD, and the NfnL proximal [4Fe-4S] cluster. The proximal [4Fe-4S] cluster signal was consistent with a fast-relaxing iron–sulfur cluster, losing signal above 30 K, in contrast with the underlying distal cluster signal, which relaxes more slowly and is observable up to 60 K. A reference sample consisting of 100 μM NfnL with 1 mM dithionite (“DTH”) was measured and further confirmed the proximal [4Fe-4S] cluster signal identity (10x DTH; Figure 2B). At a temperature of 100 K, the radical signal was stable and remained visible even up to 230 K, where the higher temperature allowed the sample to anneal halfway back to starting conditions over the course of 45 min, nearly returning to the preillumination spectrum after ∼ 5 min at 250 K (*t*_1/2_ ≥ 30 min; Figure S7). Not only was this radical longer lived than anticipated, but it also had well-behaved signal decay kinetics over the course of 5 h at 230 K, indicative of a complex mechanism of electron recombination within the low potential pathway, particularly between the radical and proximal [4Fe-4S] cluster (Figure S8, Table S4).

**Figure 2:**
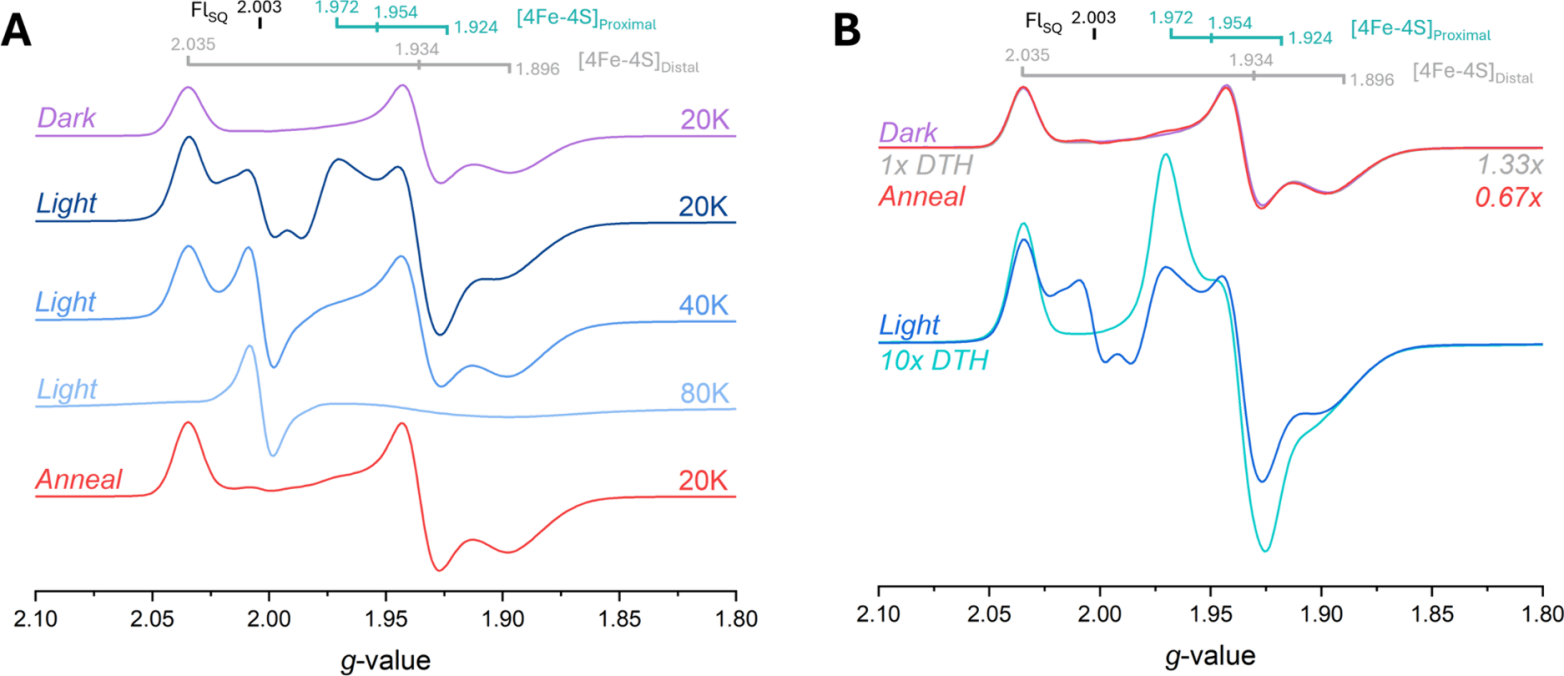
233 K illumination of NfnL in the presence of 8.8 mM NADPH with 2 W 405 nm for 1 h reveals two new species not seen in the preillumination spectrum. Power and modulation amplitude used for these data were set to 10 mW and 10 G, respectively. Panel **A** shows the characteristic distal [4Fe-4S] cluster before illumination (*Dark*; lavender) with *g*-values of 2.035, 1.934, and 1.896. Illumination with 405 nm light reveals two new species with distinct temperature dependencies (*Light*; navy, blue, light-blue) corresponding to the proximal [4Fe-4S] cluster (*g*-values of 1.972, 1.954, and 1.924) only observable at 20 K and below, and an ASQ radical (*g*_iso_ = 2.0033) observable up to 230 K (Figures S4–S7). Upon annealing the sample’s temperature to 250 K for ≤ 5 min (Figure S7), the sample was able to nearly return to the initial *Dark* condition (*Anneal*; red). Panel **B** shows the 20 K photoexcitation data from panel **A** overlaid with dithionite (DTH) references to confirm the identities of paramagnetic species. The normalized *1x DTH* (gray) data overlaps nearly identically to the *Dark* and *Anneal* spectra when corrected for intensity differences. With the exception of an organic radical at *g* = 2.0033, the *10x DTH* (cyan) spectrum is consistent with the proximal [4Fe-4S] cluster *g*-values observed in the illuminated samples, confirming the assignment of the 20 K *Light* (blue) spectra.

Illuminating oxidized NfnL alone without any NADPH generated only 1% of the total spectral intensity observed in Figure 2. Further, NfnL, FAD, or NADPH on their own was unable to reproduce the photogenerated species (Figures S9–S11). These control experiments suggested that the photogenerated species originate from the interaction between NADPH and the L-FAD in NfnL and require an oxidized L-FAD to absorb an ∼ 405 nm photon (Figure S12). Additionally, after 1 h of illumination at 233 K with 405 nm, no apparent photodegradation was observed for NfnL alone, implying the photogenerated species are part of a nondestructive and catalytically relevant pathway in NfnL (Figures S13–S15).

We assigned the observed photogenerated radical identity as an ASQ, based on its line width,^24,27–29^ line shape,^24–26,28^ spin–spin interaction with the proximal [4Fe-4S] cluster (Figures 2, S6), and the consistency with previous TAS data.^11,13^ We compared the photogenerated radical’s peak-to-peak line width to the established neutral semiquinone (NSQ) signal observed on the S-FAD located in the full NfnSL complex (Figure 1A,C).^11^ As shown in Figure 3, the photoexcitated sample generated a flavin radical signal with a peak-to-peak line width of 13.4 G at its narrowest (modulation amplitude at 1 G), in contrast to the narrowest S-FAD NSQ line width of 25.0 G, slightly broader than typical NSQ line widths of 18 to 20 G^29,30^ due to spin–spin coupling between the [2Fe-2S] and the NSQ.^11,31,32^ The large difference in peak-to-peak line width between the two radicals confirms the photogenerated radical is distinct from the S-FAD NSQ, and the 13.4 G line width is consistent with previous ASQ assignments.^24,27,28,33^ The ASQ radical also exhibited minor, but consistent anisotropy, aka “wings”, flanking the main isotropic signal seen in Figure 3. The “wings” were consistent in all tested spectral parameters (temperature, power, modulation amplitude), suggesting the radical “wings” are a feature of a single radical species and not from two different overlapping radical species.^24–26^ Figure 3B depicts the normalized, overlaid spectra of the photogenerated radical taken at the listed temperatures, showing only one species is apparent up to a temperature of 200 K.

**Figure 3:**
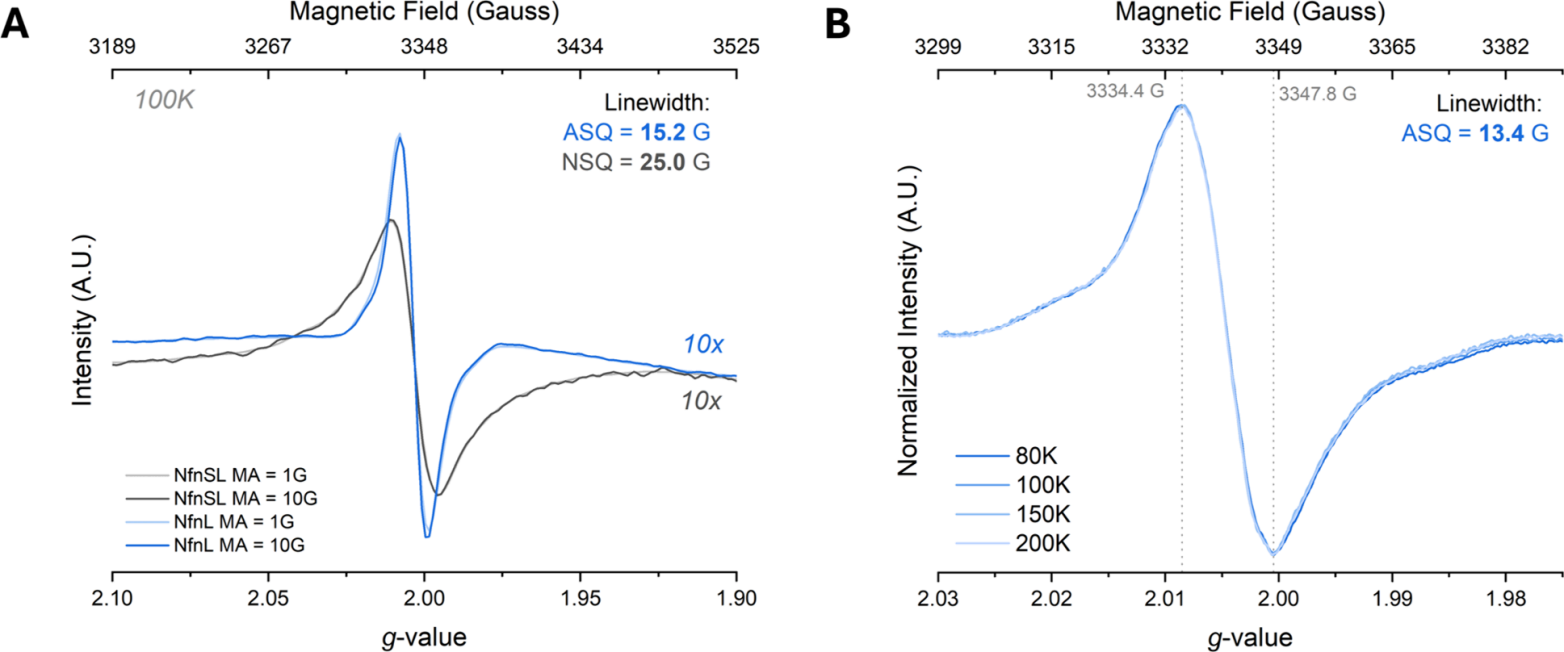
Photogenerated radical line width is consistent with an ASQ and inconsistent with an NSQ. Direct comparison of peak-to-peak line widths for the photogenerated flavin radical (blue traces) to the NSQ in the S-FAD of NfnSL (charcoal traces). Panel **A** shows the full radical spectra, with reference lines showing the peak measurements. Reducing the modulation amplitude from 10 to 1 G did not narrow the peak-to-peak line width for the NSQ, but did however narrow the photogenerated radical line width by ∼ 2 G. Panel **B** depicts normalized spectra of the observed radical species with a line width of 13.4 G at a modulation amplitude of 1 G over multiple temperatures (80 to 200 K) and shows the radical “wings” are part of the main radical feature and not from an underlying unique radical species.^24–26^ Panel **B** swept 100 G on either side of the 3350 G center field, from 3250 to 3450 G, resulting in high resolution of the radical species. The microwave power was 10 mW for panels **A** and **B**, the temperature was 100 K for panel **A**, and modulation amplitude is explicit for each trace, for which the intensities have been corrected for. Illumination at 405 nm was 90 min for the photoexcited sample spectra.

To assess the order in which the paramagnetic signals appeared in response to illumination, a time course was conducted, probing both *g* ∼ 2 and *g* ∼ 4 regions at temperatures of 3.6, 20, and 100 K. As seen in Figure 4, with increasing illumination time, the low potential pathway becomes populated with singly reduced paramagnetic species. Within the first 10 min of illumination, the radical is reduced immediately, preceding the reduction of both the proximal [4Fe-4S] cluster and distal [4Fe-4S] cluster (Figure S16). After 10 min, the constant illumination conditions led to complex rates of formation for the paramagnetic species, although these species all increased in intensity as the illumination time increased (Figure S16). After 90 min of illumination, the total signal intensity corresponding to the distal [4Fe-4S] cluster appeared to be roughly double what it was at the beginning, implying that a noteworthy portion of the preilluminated NfnL sample had no reduced distal [4Fe-4S] cluster (Figure 4A). This increase in distal [4Fe-4S] cluster intensity underlies the emergence of the radical and reduced proximal [4Fe-4S] cluster and increased with longer illumination times.

**Figure 4:**
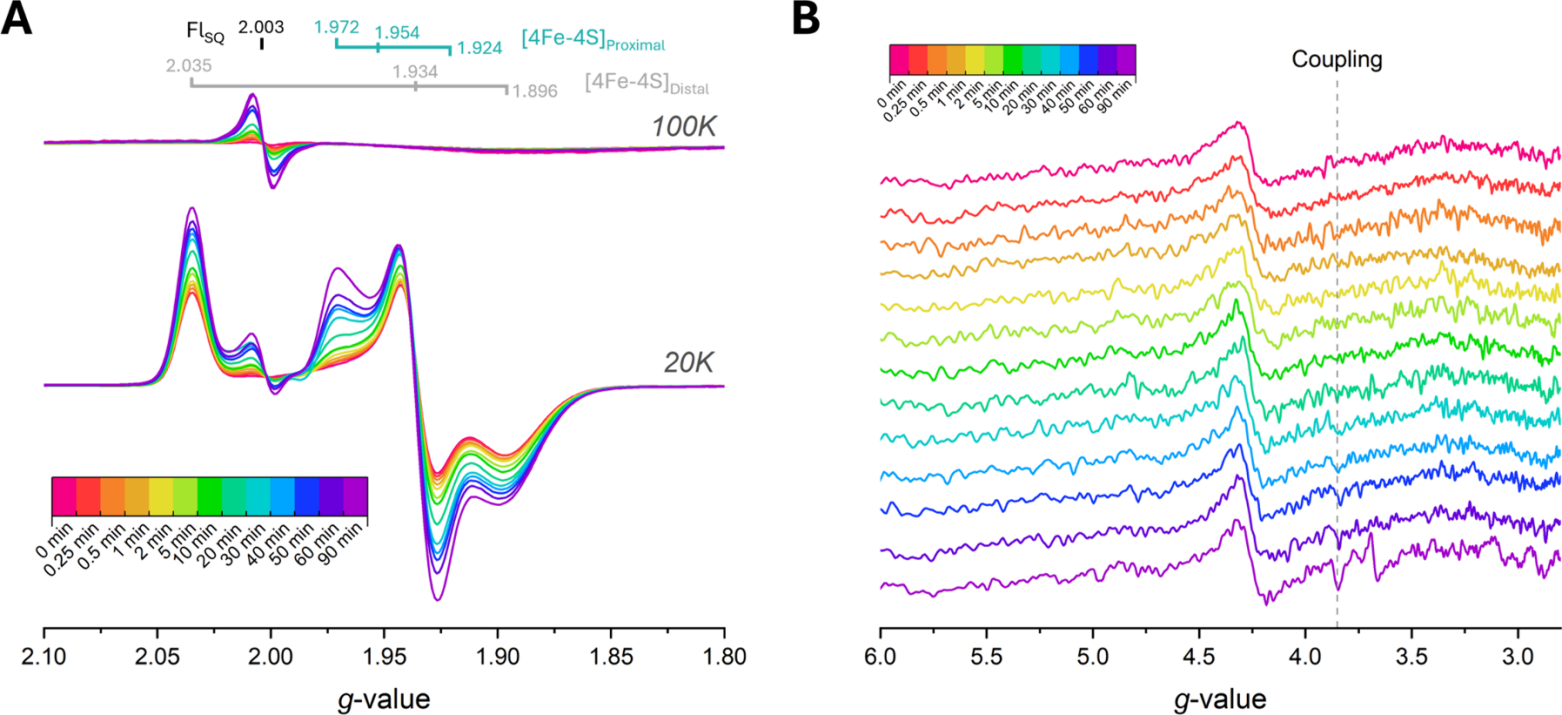
Illumination of NfnL with NADPH shows growth of both radical and proximal [4Fe-4S] cluster signals over the course of 90 min. Panel **A** depicts the overlaid spectra corresponding to the increase in the radical signal at 100 K (*above*) and the proximal [4Fe-4S] cluster signal at 20 K (*below*). In addition to the ASQ and proximal [4Fe-4S] clusters, there is an increase in the overall distal [4Fe-4S] cluster signal intensity as well. Panel **B** shows the low-field region at 3.6 K, where a signal at *g* ∼ 3.89 arises with increased illumination time (30+ min), due to the interaction between the reduced distal and proximal [4Fe-4S] clusters.^34^ The aberrant signal at *g* ∼ 3.7 for the 90 min low-field spectrum in **B** is a spectral artifact of unknown origin and likely unrelated to the experimental results (Figure S17). Spectra parameters: **A** = 10 mW power, 10 G modulation amplitude, 20 and 100 K; **B** = 10 mW power, 10 G modulation amplitude, 3.6 K.

In addition to the intensity increases in the *g* ∼ 2 region, we observed coincident increases in the intensity of a signal at *g* ∼ 3.89 indicative of spin–spin coupling between the proximal and distal [4Fe-4S] clusters once the proximal [4Fe-4S] cluster was sufficiently reduced (Figure 4B; *vide infra*). These spectral observations were repeatable between samples as well as with the same protein sample, underscoring that this process is consistent between different preparations and can regenerate the starting material. The feature at *g* = 3.7 in Figure 4B is likely a spectral artifact (Table S1, Figures 2 and S17). An extended storage period of 24 days for the 90 min phototreated sample in Figure 4 validated that the photoinduced intermediates were stable in liquid nitrogen (∼77 K) without any significant signal decay (Figure S17).

In order to see the maximal signals possible, the NADPH-treated NfnL sample was subjected to an extended illumination of 4.5 h, with the resulting spectra seen in Figure 5. After illumination, the photogenerated species were replicated with the greatest intensities observed so far, accounting for roughly 53% of the NfnL having an ASQ and showing nearly double-to-triple the previous amounts seen for the radical and proximal [4Fe-4S] cluster species (Figures 5 and S18). The overall intensity of the proximal [4Fe-4S] cluster in the 4.5 h illuminated sample was proportional to the 10x DTH reference sample (Figure 5B versus Figure 2B), showing the illumination process can achieve comparably low potentials to that of excess DTH at pH 8.8. Additionally, the observance of extra isotropic intensity flanking the main ASQ feature at 20 K (Figure 5A) indicated a spin–spin (*vide infra*; *S* = 1/2 + 1/2 = 1) interaction^31^ between the proximal [4Fe-4S] cluster and the ASQ radical, further providing evidence for low-potential pathway reduction with a flavin radical rather than an exogenously produced radical (Figure S6).

**Figure 5:**
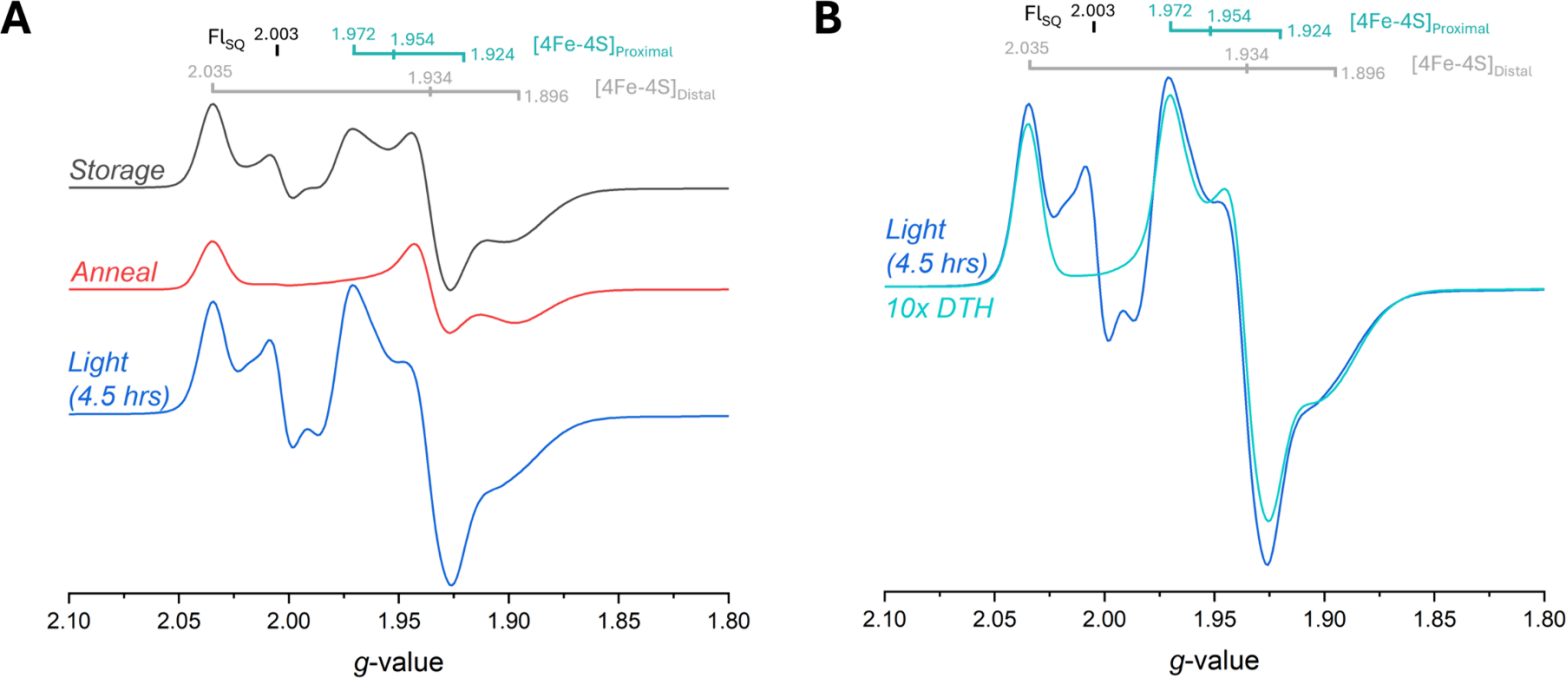
4.5 h illumination reveals large radical and proximal [4Fe-4S] cluster signals. Panel **A** shows the *Storage* (charcoal), *Anneal* (red), and *Light* (blue) conditions at 20 K to see relevant paramagnetic species. *Storage* spectrum reflects the 90 min illuminated conditions for the 2nd phototitration experiment (Figure S17) after 13 days of storage in liquid nitrogen. The *Anneal* spectrum was taken after melting the sample at room temperature before refreezing in liquid nitrogen, showing regeneration of the preillumination condition. *Light* spectrum was taken after 4.5 h of illumination at 405 nm and shows much stronger signals for the radical and proximal [4Fe-4S] cluster species, replicating the initial photochemical experiment in Figure 2 with greater intensity. Panel **B** depicts the *Light* spectrum in **A** at 20 K in comparison to that of the 10x DTH reference sample (cyan). The intensity of the proximal [4Fe-4S] cluster signal appears to have doubled or tripled in the 4.5 h experiment relative to the maximal signal observed in previous illumination spectra.

### Low-Field Cluster-to-Cluster Coupling Signal Observed When Both [4Fe-4S] Clusters are Reduced

While acquiring the illumination data, we consistently observed a low-field, semianisotropic feature located at *g* ∼ 3.89 in multiple samples, but only once both the distal [4Fe-4S] cluster and proximal [4Fe-4S] cluster were reduced (Figures 4B and 6). This *g* ∼ 3.89 feature was notably absent in the preillumination, annealed, and 1x DTH reference NfnL samples, but was present in variable intensity for any sample with significant proximal [4Fe-4S] cluster signal, especially for the 10x DTH reference sample and the 4.5 h illumination sample (Figures 6A and S19). This *g* ∼ 3.89 signal dependence on the proximal [4Fe-4S] cluster strongly implies this feature is a *Δm*_*S*_ = 2 transition stemming from triplet state coupling between the two *S* = 1/2 spins (*S*_tot_ = 1/2 + 1/2 = 1) located on the iron–sulfur clusters, especially since they are only 7.4 Å edge-to-edge away (Figure 1B).^34–36^ Further, the *g* ∼ 3.89 signal-to-noise ratio was best resolved at 7 K but quickly became weak due to the rapidly relaxing character of the signal (Figure 6B). Altogether, the *g* ∼ 3.89 coupling signal reflects a minor but important electromagnetic communication between the two reduced [4Fe-4S] clusters and may be indicative of a directional preference in electron transfer within the low-potential pathway.

**Figure 6:**
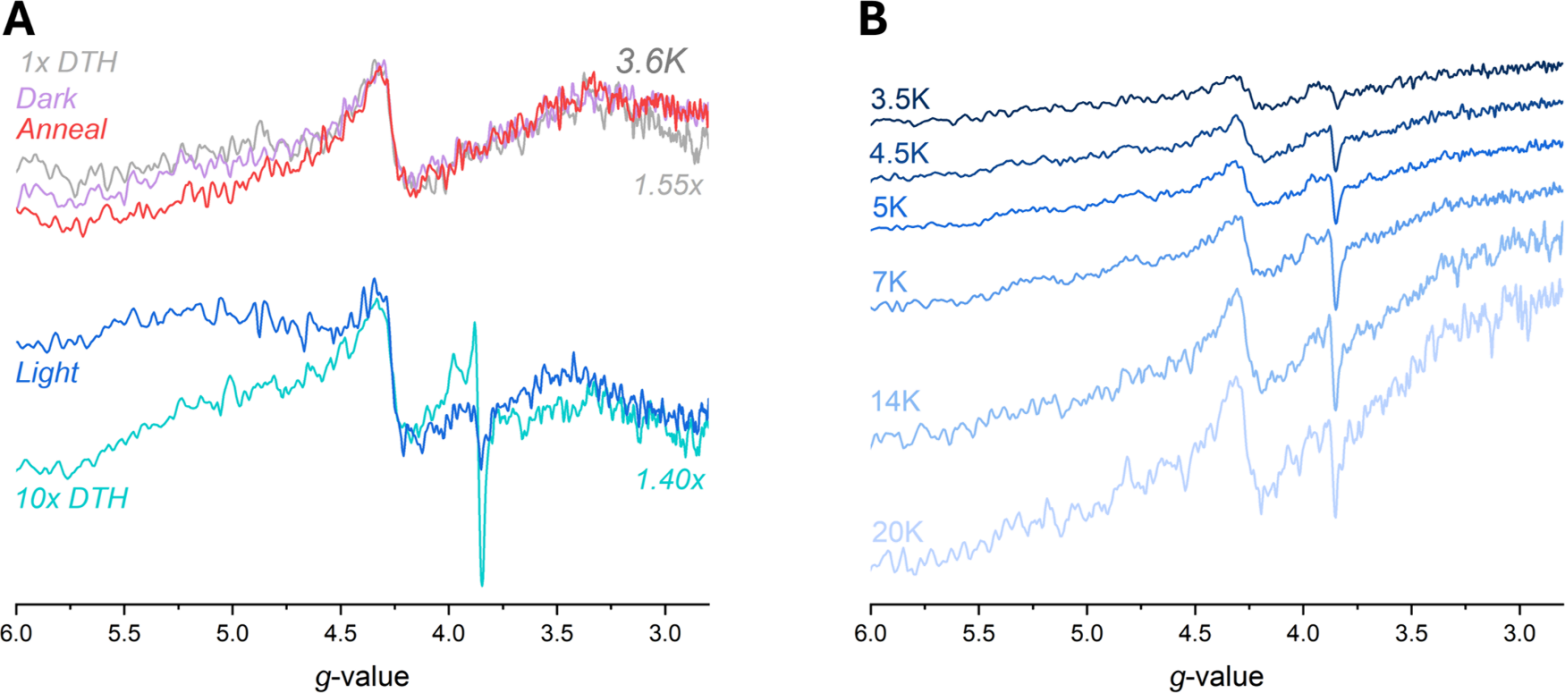
Low-field signals arising from reduced [4Fe-4S] cluster–cluster interactions. Panel **A** depicts the low field data corresponding to Figure 2A, showing the *g* ∼ 3.89 feature arises only in the samples with significant amounts of proximal [4Fe-4S] cluster, whereas the feature is absent in samples without proximal [4Fe-4S] cluster reduction (Figure S19). The *1x DTH* (gray) and *10x DTH* (cyan) values were normalized to match the “junk iron” signal at *g* ∼ 4.3 in the *Dark* (lavender) and *Light* (blue) spectra. Panel **B** shows the Curie corrected (Signal*Temperature) temperature dependence for the low field signal corresponding to the 4.5 h illumination sample in Figure 5, with the best signal-to-noise at 7 K. Spectral parameters were 10 mW microwave power, modulation amplitude 10 G (panel **A**) or 6 G (panel **B**), and temperatures as specified in each graph.

## Discussion

### Interpretating the Significance of the High Energy Intermediates

The photoexcitation method utilized here allows for use of a nondegenerative, *in situ* process that is sufficient to reduce low-potential intermediates that are unstable and difficult to characterize otherwise. In comparison with the 1x and 10x DTH references, the photogenerated proximal [4Fe-4S] cluster can be clearly identified on top of the preexisting distal [4Fe-4S] cluster signal, and based on line width, temperature, and annealing behavior data, the radical is consistent with an ASQ.

Due to the significant spectral overlap of the three iron–sulfur cofactors found in NfnSL and the difficulty in fully populating the low-potential proximal [4Fe-4S] cluster during the former *g*-value assignments, the initially reported values described a more conventional rhombic shape with *g*-values of 2.035, 1.955, 1.916.^11,13^ In this report, the proximal cluster consistently and repeatedly best fit a semiaxial shape with *g*-values of 1.972, 1.954, and 1.924, and was unexpected considering typical [4Fe-4S] cluster lineshapes are more commonly rhombic in nature.^37,38^ Both reduction by DTH and the photoexcitation method produced the proximal cluster line shape, showing the signal is an accurate representation of the true paramagnetic species and not an artifact of DTH. These two sets of *g*-value parameters were compared and fit to different accuracies, where the semiaxial parameters fit significantly better than the previously reported values, indicating this new semiaxial set is more representative of the proximal [4Fe-4S] cluster’s line shape (Figures S3–S6). The signal line shape, and ultimately the electronic structure, of the proximal [4Fe-4S] cluster in NfnL is unique relative to other site-differentiated iron sulfur clusters observed previously,^39–41^ particularly in reference to those with Fe–O ligations.^42–46^ These results show the proximal [4Fe-4S] cluster has an unconventionally compact *S* = 1/2 line shape in X-band (∼9.4 GHz) EPR.^47^ The signal shape of the proximal [4Fe-4S] cluster likely stems from the glutamic acid interaction with the Fe, leading to less electron delocalization over the mixed valence Fe^2.5+^ face resulting in a more electronically isolated spin system.^47–49^ Alternatively, the unique line shape for the proximal [4Fe-4S] cluster could be due to magnetic interactions between the two reduced [4Fe-4S] clusters, and that if there were no distal cluster signal at all then the proximal cluster would be much more rhombic in shape.^34,50^ Although significant magnetic coupling between the two [4Fe-4S] clusters is plausible, this situation is unsupported by the low temperature data which shows that neither the proximal [4Fe-4S] cluster or the distal [4Fe-4S] cluster have any major line shape splitting at lower temperatures,^50^ nor does the low field indicate any single spin system higher than *S* = 1/2 (Figure 6).

We have assigned the photogenerated radical species as an ASQ due to the numerous similarities with previously characterized ASQ species,^24,27,28,33^ the identification of an ASQ at this site in other photoexcitation experiments (TAS), and its inconsistency with the broad NSQ signal observed in the NfnS S-FAD (Figure 3).^11,13^ This radical signal completely disappears when the sample is warmed above ∼240 K without any byproduct generation or apparent damage to the protein (Figures 2A, S7, S13, S17), and similarly appears in very minor quantities in NfnL when saturated with NADPH (Figure 2).^13,33^ Considering these attributes, as well as the reversibility and coaccumulation of the radical and the proximal [4Fe-4S] cluster, we have assigned this ASQ as a catalytically relevant species in the mechanism of electron bifurcation. The illumination control experiments showing the very minor radical and distal [4Fe-4S] cluster signals (∼1% relative to NADPH samples) formed in the oxidized NfnL control further support the relevance of the NADPH (Figures S9–S11, S14, S15). Over the course of 1 h of illumination, the free FAD control photoreduced to the HQ state without evidence of a radical *in situ* (Figures S14, S15). This photoactivity of free FAD further solidified the mechanistic relevance of NADPH binding near the L-FAD, as without NfnL or NADPH, there is no significant accumulation of any radical species, displaying the ASQ radical is facilitated *specifically* by the nearby environment surrounding the flavin in NfnL. For the free FAD control to be reduced at pH 8.8 without exogenous chemical reductants, the electrons are either intramolecularly sourced^51–53^ or pulled from the buffer solution, possibly from HEPES itself, as it has a minor amount of redox activity.^54–56^ The *in situ* photoreduction of the FAD control suggests that the buffer solution is the source of the minor reduction (1%) of the photoexcited L-FAD observed in the oxidized NfnL control (Figures S9–S11).

The observation of an anisotropic low-field (∼1600 G; *g* ∼ 3.89) signal at low-temperatures (T_opt_ ∼ 7 K) in reduced NfnL was assigned as iron–sulfur cluster spin–spin coupling and is consistent with the edge-to-edge distance between the proximal and distal [4Fe-4S] clusters of about 7.4 Å (Figure 1B). This low-field signal is emergent from the spin system having *S* = 1/2 + 1/2 = 1, and as this *S* = 1 signal depends on both clusters, it is primarily dependent on the reduction of the proximal [4Fe-4S] cluster.^34–37^ This is validated by the results in Figure 6A, showing that the most intense *g* ∼ 3.89 features occur with the most intense proximal cluster signals and are completely absent when no proximal [4Fe-4S] cluster is observed. This coupling signal is an insight into the finely tuned intercommunication between the downstream distal [4Fe-4S] cluster and the intermediate proximal [4Fe-4S] cluster. This through space coupling between the clusters may be specifically tuned for electron transfer reversibility as well, especially since annealing the sample at higher temperatures reestablishes the original equilibrium (Figure 2).^57–60^

### The Mechanism of Photoexcited Electron Transfer

Flavin photoexcitation is a well-studied process where the oxidized state generates a transient superoxidizing flavin by exciting an electron within a singlet-state electron pair to an excited triplet-state from the HOMO to the LUMO.^61,52,53,62,63^ This excited state oxidizes a nearby electron donor to fill the newly unoccupied lower energy state, producing a singly reduced semiquinone. In our experiment, the electron source is the nearby NADPH.^11^ This photoinduced mechanism of ASQ generation in free flavin is well understood and essentially only operates via single electron chemistry.^63^

The mechanism of photoexcited electron transfer in NfnL is hypothesized to generate an ASQ that is capable of transferring its electron to the proximal [4Fe-4S] cluster and to the distal [4Fe-4S] cluster (if it is oxidized; Figure 7). The injection of 2 electrons into the low-potential pathway is possible since NADPH is a two-electron donor and could feasibly provide both electrons to the low-potential pathway. If the ASQ is photogenerated from a nearby NADPH, then a highly unstable NADP^•^ would be coincidently formed. As we do not see any obvious evidence of an additional pyridine nucleotide radical (e.g., “NADP^•^”)^64^ in the photogenerated spectra (Figures 3 and S6), we can conclude that there must be a rapid 2 electron mechanism that produces the ASQ which transfers to the proximal [4Fe-4S] cluster at 233 K. Literature on the single electron chemistry of NAD­(H) calculates the first midpoint reduction potential of NAD to NAD^•^ as −940 mV vs SHE, with the second midpoint reduction potential of NAD^•^ to NADH as +300 mV vs SHE.^65^ These large single electron redox potentials imply that once one electron has been stripped from the nearby NADPH substrate by the photoexcited L-FAD*, the reducing power of the NADP^•^ facilitates rapid electron transfer within the low-potential pathway to repopulate the ASQ on the L-FAD while simultaneously reducing the proximal [4Fe-4S] cluster.

**Figure 7:**
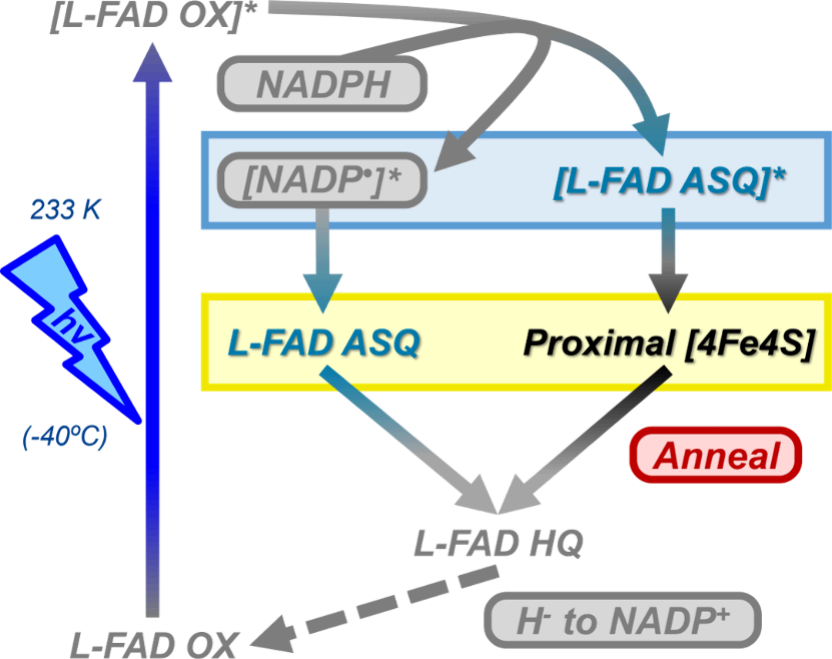
Hypothesized photogeneration mechanism for the observed species based on the data presented in this report. Starting in the oxidized state (*bottom left*), the L-FAD is excited by 405 nm light (*left, blue*), producing a highly transient superoxidizing flavin state (*top left*). This highly oxidizing flavin strips an electron from the nearby NADPH (Figure S20) to transiently reduce the L-FAD to the ASQ state (*top right*), producing an unobserved NADP^•^ radical (asterisks, *blue box*). This transient ASQ rapidly transfers its electron down the low-potential pathway to the proximal [4Fe-4S] cluster while being immediately rereduced by the NADP^•^ radical to produce the main paramagnetic intermediates observed in this report (*yellow box*). Once the sample is brought above 230 K, the sample is able to recombine the electron pair to form the lower-energy diamagnetic L-FAD HQ state observed in UV–visible experiments and ultimately regenerate the resting equilibrium present at the beginning of the experiment.^13^ If the distal [4Fe-4S] cluster is already reduced preceding this photogeneration process, then all 3 redox cofactors are paramagnetic and the coupling between the proximal and distal [4Fe-4S] clusters is observable.

### Comparison of Photoexcitation Methods

Significant accumulation of an unstable ASQ is challenging to accomplish in TAS experiments due to spectral overlap with NADPH from 340 to 370 nm and at room temperature (293 K).^11,13^ Not only is the accumulation of the ASQ difficult but also the proportionally small absorbance changes for [4Fe-4S] clusters make tracking ET between the proximal [4Fe-4S] cluster and the ASQ difficult to assess by TAS. The main distinction between the TAS experiment and this study is the constant 405 nm illumination used to excite the L-FAD, as opposed to the pulsed photoexcitation in TAS, which results in continued accumulation of low-potential pathway intermediates (Figure S16). Regardless, the method used in this study is proposed to utilize the same initial photoexcitation mechanism as the TAS experiment that targets and requires the oxidized L-FAD to produce (and constantly accumulate) the ASQ intermediate (Figure S12), contributing to the refined model depicted in Figure 7. Consistent with the TAS experimental mechanism, the oxidized L-FAD in NfnL absorbs a 405 nm photon and becomes photoexcited to form the highly transient superoxidizing flavin species (“[L-FAD OX]*”).^11,13,66^ The superoxidizing flavin can then accept an electron from the nearby NADPH donor to produce an ASQ (Figure S20). After the initial transfer, the highly reducing NADP^•^ provides sufficient pressure to push further downstream reduction of the proximal [4Fe-4S] cluster as the NADP^•^ simultaneously re-reduces the L-FAD to the ASQ to produce the observed results in this report (Figures S6 and S16). If the distal [4Fe-4S] cluster is already reduced, as some initially are in the presence of NADPH (Figure 2), then the NfnL has unpaired electrons located on all 3 redox cofactors and exhibits the coupling signal between the proximal and distal [4Fe-4S] clusters, located at *g* ∼ 3.89 (Figure 6). However, if the distal [4Fe-4S] cluster is in the oxidized form, there is an additional intramolecular electron transfer from the proximal [4Fe-4S] cluster to the distal [4Fe-4S] cluster (Figure S16), leaving just a radical on the flavin as an ASQ along with the reduced distal [4Fe-4S] cluster (Figure S10A).

### Advancing a More Robust Model of Electron Bifurcation

By utilizing photoexcitation to initiate electron transfer at cryogenic temperatures, we were able to populate electrons on all of the cofactors in NfnL, including the accumulation of the unstable ASQ species at the L-FAD active site. The resulting EPR data provide insights into the interactions between the low potential pathway cofactors, which have been difficult to ascertain before now due to the inability to visualize the ASQ and proximal [4Fe-4S] cluster simultaneously and with high yields. Because these intermediates are visible and enrichable, we can observe the downhill electron transfer order from the ASQ to the proximal [4Fe-4S] cluster to the distal [4Fe-4S] cluster. Once these reduced states are produced, electron recombination at higher temperatures reveals critical insights into the electron transfer mechanism of confurcation (i.e., the reverse of bifurcation) in NfnL, which are otherwise extremely difficult to visualize. The observation of coupling, notably the low field *g* = 3.89 feature between the proximal and distal [4Fe-4S] clusters as well as extra *g* ∼ 2 intensity flanking the radical at *g* = 2.0033 due to ASQ and proximal [4Fe-4S] cluster interaction, suggests the low-potential pathway has a network of through-space interactions that appear to prime nearby cofactors to sense pathway redox states.

Comparing the results here with photoexcitation performed at room temperature (i.e., TAS) highlights a key difference in the stability and accumulation of ASQ. At cryogenic temperatures, both ASQ and proximal cluster are generated and steadily increase in concentration over 4.5 h (Figure S16), while in the TAS experiment ASQ is rapidly formed but due to back electron transfer with the proximal cluster it has a lifetime of only ∼10 ps. Furthermore, we observed 3 main radical decay phases when the sample is warmed to 230 K (Figure S8) that suggests a more complex mechanism of electron recombination through the low-potential pathway and may include various switching mechanisms to prevent electron transfer in the confurcating direction. One possibility for this temperature dependent behavior may be movement of the Arg333 residue, which is a newly identified determinant of electron bifurcation ability.^17^ When the β-factors between the NADPH-bound and -unbound NfnSL crystal structures are compared, there are notable changes in the region corresponding to the ∼300 to ∼400 amino acid loop (Figure S21). While the R333 residue is not interacting with the L-FAD in either of the crystal structures (and likely represents the low-temperature state), there is ample room for it to move over the O2’, N3, and O4’ moieties of the L-FAD. As modeled in the crystal structure, the R333 interacts with two glutamic acid residues, E335 and E336 (Figure S22). At room temperature, there may be enough conformational sampling that a change in the interaction of R333 from E335/336 to L-FAD may be possible. At cryogenic temperatures, we hypothesize that it is locked out of position (similar to its position in the crystal structure; Figure 1B and Figure S21) and when the sample is annealed it can move, affecting the reversibility of the electron transfer events across the low potential pathway and/or the stability of the ASQ (Figure S22). These results suggest a previously unobserved gate of electron transfer along the low potential pathway in NfnL that is likely critical for efficient partitioning of electrons during the electron bifurcation mechanism.

## Conclusions

The reversible photogeneration of unstable and high-energy paramagnetic intermediates described here allowed for the unambiguous observation of key electron transfer events within the low-potential pathway of Nfn. The recombination of electrons from the low-potential pathway back to the L-FAD suggested a complex and redox-sensitive switch, representing a previously unknown aspect of this elegant mechanism. These studies have described the fundamental characteristics of key bifurcation intermediates and furthered our understanding of how cofactor environments are tuned to initiate and control high-energy electron transfer. Exploiting photoexcitation further opens the door to studying more subtle mechanistic steps within bifurcation, such as proton-coupled electron transfers and the effects of additional nearby amino acid residues on electron transfer (Figures 1B, S21, S22). In addition to expanding our mechanistic understanding of Nfn, we demonstrate the utility of applying this photoexcitation method to other FBEB enzymes and flavin-involved electron transfer systems.

Understanding the physical principles that govern the mechanism of electron transfer in biology is key to building better and more robust synthetic systems capable of highly controlled electron transfer. Additionally, the photogeneration of the low-potential species observed in this report may have broader applications, such as exogenous *in vitro* reductant upconversion for industrial or biosynthetic applications, providing low-potential electrons sourced from NADPH in a controlled manner and pave a way for greater photochemical applications of this unique and thermostable enzyme.

## Materials and Methods

All materials and methods were prepared and performed anoxically unless written otherwise.

### Production of Recombinant P.f. NfnL and P.f. NfnSL in *E. coli* BL21 (DE3)

NfnL and NfnSL were grown, overexpressed, purified, and reconstituted from *Escherichia coli* (*E. coli*) as previously described.^12,13^ Previously constructed streptomycin^R^ pCDFDuet-1 plasmids containing either the *Pyrococcus furiosus* (*P.f*.; DSM 3638) *nfnL* gene with an N-terminal Strep-II polypeptide affinity tag (protein sequence GWSHPQFEK) or the *nfnS* and *nfnL* genes with an N-terminal Strep-II polypeptide affinity tag were used in this study (GenScript, USA). Since As-purified NfnL and NfnSL lack complete cofactors (8 Fe, 1 FAD for NfnL; 10 Fe, 2 FAD for NfnSL), FAD and Fe reconstitution was carried out as previously described^12,13^ to ensure enzymes were cofactor-replete.

### Protein Concentration Assessed by Rose Bengal Colorimetric Assay

Protein concentrations were quantified aerobically using an adapted version of the rose bengal dye-based colorimetric assay.^15,67,68^ For the assays, the following stock solutions were prepared: 1 mg/mL rose bengal dye solution, 50% acetic acid quenching solution, and 10 mM potassium phosphate (KP_i_) at pH 7.0. The setup for the rose bengal colorimetric assay was as follows: Add 1800 μL of 10 mM KP_i_ minus the volume of intended protein sample (2–20 μL), to which the protein sample volume (2–20 μL) was added. To begin the assay, 100 μL of the 1 mg/mL rose bengal solution was added to the 1800 μL protein solution. The protein-dye mixture was allowed to react for 3 min. After the incubation time, the protein-dye mixture was quenched with 100 μL of the 50% acetic acid solution (final mixture volume is 2000 μL), and a UV–visible spectrum focused on 560 nm was taken within 5 min of acid-quenching. Finally, the sample-of-interest’s absorbance was compared to a BSA standard curve (11 points; 0 – 20 μg of BSA, increments of 2 μg) to assess protein concentration in sample-of-interest in μg/mL and corrected for any dilutions.

### Cofactor Quantitation of NfnL and NfnSL

Cofactor reconstitution was assessed aerobically by iron-counting as previously described,^13,12,69,70^ as well as flavin-counting as previously described.^12,13,71^ For NfnL, iron and FAD loading was determined to be 9.0 ± 0.2 iron atoms per NfnL monomer (112 ± 2%) and 1.21 ± 0.03 FAD per NfnL monomer (121 ± 3%). For NfnSL, iron and FAD loading was determined to be 10.2 ± 0.1 iron atoms per NfnSL unit (102 ± 1%) and 1.81 ± 0.04 FAD per NfnSL unit (91 ± 2%).

### Preparation of Electron Paramagnetic Resonance Samples

Electron paramagnetic resonance (EPR) samples were prepared as outlined in Table S1 to final volumes of 220 μL with 150 mM HEPES pH 8.80, 200 mM NaCl, and 5% glycerol (“buffer”). Of the total 220 μL, 200 μL was transferred to a quartz EPR tube for spectral acquisition and photoexcitation experiments, and the remaining 20 μL was used for concurrent preillumination UV–visible measurements and protein concentration confirmation by rose bengal colorimetric assay on a Beckman Coulter DU 800 UV–visible spectrophotometer with deuterium (UV) and tungsten (visible) lamps. NADPH was sourced from Research Products International (RPI; Mt. Prospect, IL) and stock concentrations were quantified at 340 nm via UV–visible measurements using the extinction coefficient (ε_340_) of 6.22 mM^–1^ cm^–1^.^72–74^ Sodium dithionite and flavin adenine dinucleotide (FAD) were sourced from MilliporeSigma (Burlington, MA), where they were weighed prior to the preparation of stock solutions. For photoexcitation experiments (NfnL_PE_), NfnL concentrations were prepared to a final concentration of 140 μM with 8.8 mM NADPH final concentration. The relatively high concentration of 8.8 mM NADPH was selected to maintain consistency with previous studies^15^ and to stave off any depletion of NADPH in the case that it was subject to photodegradation by sustained 405 nm illumination. 140 μM NfnL alone, 200 μM FAD and 8.8 mM NADPH were prepared from stock solutions as photoexcitation control experiments. Reduced NfnL (NfnL_Red_) was prepared with 10x dithionite (1 mM DTH) as a reference for the proximal cluster EPR spectrum for the fully reduced state of NfnL. Partially reduced NfnL (NfnL_Dist_) was prepared with 1x dithionite (0.1 mM DTH) as a reference for the distal cluster EPR spectrum in NfnL. As-isolated NfnSL was prepared to a final concentration of 150 μM as a neutral semiquinone (NSQ) reference for flavin radical line width comparisons for organic radicals observed in this study.

### Illumination Conditions

Samples were illuminated using a custom, variable temperature cryogenic illumination system comprising a THORLABS (Newton, NJ) 4P4 – 100 mm Integrating Sphere with 4 modular faces outfitted with 2 opposing THORLABS M405L4 1000 mW minimum LED diodes for 2 W illumination at 405 nm excitation wavelength. All illumination was carried out at 233 K (−40 °C) to facilitate electron transfer^22,23^ and integrating sphere temperature was confirmed using an external K-type thermocouple (Omega HH306A) placed within an EPR tube. NfnL_PE‑I_, FAD, NADPH, and NfnL_Ox_ were all illuminated at 233 K for 1 h before spectral acquisition. NfnL_PE‑T_ was illuminated at 233 K for various times, at which spectral acquisition was taken at the conclusion of each illumination interval.

### EPR Data Acquisition, Parameters, and Data Processing

All EPR spectra were collected on an ELEXSYS E500 continuous-wave (CW) X-band (∼9.4 GHz) spectrometer (Bruker; Billerica, MA) outfitted with a super high-Q perpendicular-mode resonator and an in-cavity, cryogen-free variable temperature helium system (ColdEdge Technologies; Allentown, PA) paired to a Mercury iTC temperature controller (Oxford Instruments; Abingdon, UK). Variable parameters such as temperature, modulation amplitude (MA), and microwave power are specified for relevant traces in each figure and range from 3.5 to 230 K, 1 to 10 G, and 1 to 10 mW, respectively. Parameters applying to all collected spectra were: Modulation frequency of 100 kHz; gain of 60 dB; conversion time of 81.92 ms; 1024 points per spectrum; 5 scan averaging; field sweep 2000 G; and depending on the region of interest, center field of 3350 G (*g* ∼ 2 region) or 1400 G (*g* ∼ 4). Microwave frequency was around 9.37 GHz for all spectra, and each spectrum’s *g*-value plot was corrected by its corresponding microwave frequency recorded during spectral acquisition. EPR data was exported and initially processed using an export program that calculates *g*-values in MATLAB R2020b. Data were processed in OriginPro 2023, and *S* = 1/2 (*g* ∼ 2 region) spectra were simulated using EasySpin’s pepper function in MATLAB R2020b.^75,76^ ASQ radical spins were quantified in comparison to those of a 100 μM Cu­(II) TEA standard at a volume of 200 μL recorded at the same spectral acquisition parameters and temperatures noted for the corresponding radical spectra (Figure S18).

Protein model figures were produced using PyMOL software (The PyMOL Molecular Graphics System, Version 1.2r3pre, Schrödinger, LLC.) which were further processed by layering images in Microsoft PowerPoint. Figures with EPR data were generated by using OriginPro 2023.

## Supplementary Material



## Notes

General EPR parameters, EPR sample conditions, EPR power saturation spectra, EPR spectra simulations, ASQ stability at 230–250 K spectra + fitting, photocontrol EPR spectra, UV–vis spectra, additional phototitration data, supporting protein model figures, and supporting references (PDF)

## Abbreviations

FBEB: flavin-based electron bifurcation
e^–^: electron
ET: electron transfer
NAD^+^: oxidized nicotinamide adenine dinucleotide
NADH: reduced nicotinamide adenine dinucleotide
NADP^+^: oxidized nicotinamide adenine dinucleotide phosphate
NADPH: reduced nicotinamide adenine dinucleotide phosphate
NfnSL: NADH-dependent NADP^+^:ferredoxin oxidoreductase with both subunits
*P.f.*: *Pyrococcus furiosus*
FAD: flavin adenosine dinucleotide
L-FAD: bifurcating flavin adenosine dinucleotide present in NfnL
S-FAD: nonbifurcating flavin adenosine dinucleotide present in NfnS
Fd: ferredoxin
Fd_ox_: oxidized ferredoxin
Fd_red_: reduced ferredoxin
Fe: iron
[4Fe-4S]: cubane iron–sulfur cluster
[2Fe-2S]: dinuclear iron sulfur cluster
Ox: oxidized
SQ: semiquinone
ASQ: Anionic semiquinone
NSQ: neutral semiquinone
HQ: hydroquinone
AHQ: Anionic hydroquinone
mV: millivolts
KP_i_: potassium phosphate
BSA: bovine serum albumin
μL: microliters
mL: milliliters
μg: micrograms
mg: milligrams
μM: micromolar, or micromole per liter
mM: millimolar, or millimole per liter
EPR: electron paramagnetic resonance
TA­(S): transient absorption (spectroscopy)
ps: picosecond
DTH: dithionite, a low potential reductant
mW: milliwatts
MA: modulation amplitude
GHz: gigahertz
kHz: kilohertz
dB: decibels
ms: milliseconds
K: kelvin
G: gauss
hv: light
nm: nanometers
mm: millimiter
cm: centimeter
LED: light emitting diode
LN_2_: liquid nitrogen
SEC: spectroelectrochemistry
PCET: proton-coupled electron transfer. Cu­(II) TEA, copper­(II) triethanolamine

## References

[1] Buckel W., Thauer R. K. (2013). Energy Conservation via Electron Bifurcating Ferredoxin
Reduction and Proton/Na+ Translocating Ferredoxin Oxidation. Evol. Asp. Bioenerg. Syst..

[2] Peters J. W., Miller A.-F., Jones A. K., King P. W., Adams M. W. (2016). Electron
Bifurcation. Biocatal. Biotransformation Bioinorg.
Chem..

[3] Mitchell P. (1975). The Protonmotive
Q Cycle: A General Formulation. FEBS Lett..

[4] Mitchell P. (1976). Possible Molecular
Mechanisms of the Protonmotive Function of Cytochrome Systems. J. Theor. Biol..

[5] Herrmann G., Jayamani E., Mai G., Buckel W. (2008). Energy Conservation
via Electron-Transferring Flavoprotein in Anaerobic Bacteria. J. Bacteriol..

[6] Yuly J. L., Zhang P., Beratan D. N. (2021). Energy
Transduction by Reversible
Electron Bifurcation. Curr. Opin. Electrochem..

[7] Wise C. E., Ledinina A. E., Yuly J. L., Artz J. H., Lubner C. E. (2021). The Role
of Thermodynamic Features on the Functional Activity of Electron Bifurcating
Enzymes. Biochim. Biophys. Acta BBA - Bioenerg..

[8] Buckel W., Thauer R. K. (2018). Flavin-Based Electron
Bifurcation, A New Mechanism
of Biological Energy Coupling. Chem. Rev..

[9] Miura R. (2001). Versatility
and Specificity in Flavoenzymes: Control Mechanisms of Flavin Reactivity. Chem. Rec..

[10] Imran S. M. S., Wiley S. A., Lubner C. E. (2024). Electrochemistry of Flavin-Based
Electron Bifurcation: ‘Current’ Past and ‘Potential’
Futures. Curr. Opin. Electrochem..

[11] Lubner C. E., Jennings D. P., Mulder D. W., Schut G. J., Zadvornyy O. A., Hoben J. P., Tokmina-Lukaszewska M., Berry L., Nguyen D. M., Lipscomb G. L., Bothner B., Jones A. K., Miller A.-F., King P. W., Adams M. W. W., Peters J. W. (2017). Mechanistic Insights
into Energy Conservation by Flavin-Based Electron Bifurcation. Nat. Chem. Biol..

[12] Ortiz S., Niks D., Wiley S., Lubner C. E., Hille R. (2023). Rapid-Reaction
Kinetics of the Bifurcating NAD^+^-Dependent NADPH:Ferredoxin
Oxidoreductase NfnI from *Pyrococcus Furiosus*. J. Biol. Chem..

[13] Wise C. E., Ledinina A. E., Mulder D. W., Chou K. J., Peters J. W., King P. W., Lubner C. E. (2022). An Uncharacteristically
Low-Potential
Flavin Governs the Energy Landscape of Electron Bifurcation. Proc. Natl. Acad. Sci. U. S. A..

[14] Ortiz, S. ; Niks, D. ; Vigil, W. ; Tran, J. ; Lubner, C. E. ; Hille, R. Chapter Eighteen - Spectral Deconvolution of Electron-Bifurcating Flavoproteins. In Methods in Enzymology; Richard, J. P. , Moran, G. R. , Eds.; Academic Press, 2023; Vol. 685, pp 531–550. 10.1016/bs.mie.2023.03.011. 37245914

[15] Wiley S. A., Spackman I. J., Lubner C. E. (2026). Differential Ligation Alters Electronic
State and Coupling Signals of Iron-Sulfur Clusters in Flavin-Based
Electron Bifurcation. J. Inorg. Biochem..

[16] Wise C. E., Ledinina A. E., Lubner C. E. (2022). Site-Differentiated
Iron-Sulfur Cluster
Ligation Affects Flavin-Based Electron Bifurcation Activity. Metabolites.

[17] Huang S., Saad Imran S. M., Lanahan A. A., Hammer S. K., Lubner C. E., Lynd L. R., Olson D. G. (2025). A Distinct Class of Ferredoxin:NADP+
Oxidoreductase Enzymes Driving Thermophilic Ethanol Production. J. Biol. Chem..

[18] He Y., Barone M., Meech S. R., Lukacs A., Tonge P. J. (2025). Light-Driven
Enzyme Catalysis: Ultrafast Mechanisms and Biochemical Implications. Biochemistry.

[19] Frisell W. R., Chung C. W., Mackenzie C. G. (1959). Catalysis
of Oxidation of Nitrogen
Compounds by Flavin Coenzymes in the Presence of Light. J. Biol. Chem..

[20] Massey V., Stankovich M., Hemmerich P. (1978). Light-Mediated Reduction of Flavoproteins
with Flavins as Catalysts. Biochemistry.

[21] Williams-Smith D. L., Heathcote P., Sihra C. K., Evans M. C. W. (1978). Quantitative
Electron-Paramagnetic-Resonance Measurements of the Electron-Transfer
Components of the Photosystem-I Reaction Centre. Biochem. J..

[22] Chica B., Ruzicka J., Pellows L. M., Kallas H., Kisgeropoulos E., Vansuch G. E., Mulder D. W., Brown K. A., Svedruzic D., Peters J. W., Dukovic G., Seefeldt L. C., King P. W. (2022). Dissecting
Electronic-Structural Transitions in the Nitrogenase MoFe Protein
P-Cluster during Reduction. J. Am. Chem. Soc..

[23] Vansuch G. E., Mulder D. W., Chica B., Ruzicka J. L., Yang Z.-Y., Pellows L. M., Willis M. A., Brown K. A., Seefeldt L. C., Peters J. W., Dukovic G., King P. W. (2023). Cryo-Annealing of
Photoreduced CdS Quantum Dot-Nitrogenase MoFe Protein Complexes Reveals
the Kinetic Stability of the E4­(2N2H) Intermediate. J. Am. Chem. Soc..

[24] DeRose V. J., Woo J. C. G., Hawe W. P., Hoffman B. M., Silverman R. B., Yelekci K. (1996). Observation of a Flavin
Semiquinone in the Resting
State of Monoamine Oxidase B by Electron Paramagnetic Resonance and
Electron Nuclear Double Resonance Spectroscopy. Biochemistry.

[25] Hyde J. S., Göran Eriksson L. E., Ehrenberg A. (1970). EPR Relaxation
of Slowly Moving Flavin Radicals: “Anomalous” Saturation. Biochim. Biophys. Acta BBA - Gen. Subj..

[26] Ehrenberg A. (1960). Detailed ESR Spectra
of the Free Radicals of FMN and FAD. Acta Chem.
Scand..

[27] Palmer G., Müller F., Massey V. (1971). Electron Paramagnetic Resonance Studies
on Flavoprotein Radicals. Flavins Flavoproteins.

[28] Edmondson D. E., Ackrell B. A. C., Kearney E. B. (1981). Identification of Neutral and Anionic
8α-Substituted Flavin Semiquinones in Flavoproteins by Electron
Spin Resonance Spectroscopy. Arch. Biochem.
Biophys..

[29] Nohr, D. ; Weber, S. ; Schleicher, E. Chapter Ten - EPR Spectroscopy on Flavin Radicals in Flavoproteins. In Methods in Enzymology; Palfey, B. A. , Ed.; Academic Press, 2019; Vol. 620, pp 251–275. 10.1016/bs.mie.2019.03.013. 31072489

[30] Medina M., Gomez-Moreno C., Cammack R. (1995). Electron Spin Resonance and Electron
Nuclear Double Resonance Studies of Flavoproteins Involved in the
Photosynthetic Electron Transport in the Cyanobacterium Anabaena Sp.
PCC 7119. Eur. J. Biochem..

[31] Stevenson R. C., Dunham W. R., Sands R. H., Singer T. P., Beinert H. (1986). Studies on
the Spin-Spin Interaction between Flavin and Iron-Sulfur Cluster in
an Iron-Sulfur Flavoprotein. Biochim. Biophys.
Acta BBA-Protein Struct. Mol. Enzymol..

[32] Mohsen A.-W. A., Rigby S. E. J., Jensen K. F., Munro A. W., Scrutton N. S. (2004). Thermodynamic
Basis of Electron Transfer in Dihydroorotate Dehydrogenase B from
Lactococcus Lactis: Analysis by Potentiometry, EPR Spectroscopy, and
ENDOR Spectroscopy. Biochemistry.

[33] Schut G. J., Mohamed-Raseek N., Tokmina-Lukaszewska M., Mulder D. W., Nguyen D. M. N., Lipscomb G. L., Hoben J. P., Patterson A., Lubner C. E., King P. W., Peters J. W., Bothner B., Miller A.-F., Adams M. W. W. (2019). The
Catalytic Mechanism of Electron-Bifurcating
Electron Transfer Flavoproteins (ETFs) Involves an Intermediary Complex
with NAD+. J. Biol. Chem..

[34] Mathews R., Charlton S., Sands R. H., Palmer G. (1974). On the Nature of the
Spin Coupling between the Iron-Sulfur Clusters in the Eight-Iron Ferredoxins. J. Biol. Chem..

[35] Duggan D. M., Hendrickson D. N. (1974). Magnetic
Exchange Interactions in Transition Metal
Dimers. III. Nickel­(II) Di-.Mu.-Cyanato, Di-.Mu.-Thiocyanato, and
Di-.Mu.-Selenocyanato Complexes and Related Outer-Sphere Copper­(II)
Complexes. Inorg. Chem..

[36] Salerno J. C., Ohnishi T., Lim J., Widger W. R., King T. E. (1977). Spin Coupling
between Electron Carriers in the Dehydrogenase Segments of the Respiratory
Chain. Biochem. Biophys. Res. Commun..

[37] Mouesca J.-M., Lamotte B. (1998). Iron-Sulfur Clusters and Their Electronic and Magnetic
Properties. Coord. Chem. Rev..

[38] Hagen, W. R. EPR Spectroscopy of Iron—Sulfur Proteins. In Advances in Inorganic Chemistry; Cammack, R. , Ed.; Academic Press, 1992; Vol. 38, pp 165–222. 10.1016/S0898-8838(08)60064-1.

[39] Bak D. W., Elliott S. J. (2014). Alternative FeS
Cluster Ligands: Tuning Redox Potentials
and Chemistry. Biocatal. Biotransformation Bioinorg.
Chem..

[40] Tiago
de Oliveira F., Bominaar E. L., Hirst J., Fee J. A., Münck E. (2004). Antisymmetric Exchange in [2Fe-2S]­1+ Clusters: EPR
of the Rieske Protein from Thermus Thermophilus at pH 14. J. Am. Chem. Soc..

[41] Lubner C. E., Artz J. H., Mulder D. W., Oza A., Ward R. J., Williams S. G., Jones A. K., Peters J. W., Smalyukh I. I., Bharadwaj V. S., King P. W. (2022). A Site-Differentiated
[4Fe-4S] Cluster
Controls Electron Transfer Reactivity of Clostridium Acetobutylicum
[FeFe]-Hydrogenase I. Chem. Sci..

[42] Tao L., Zhu W., Klinman J. P., Britt R. D. (2019). Electron Paramagnetic Resonance Spectroscopic
Identification of the Fe-S Clusters in the SPASM Domain-Containing
Radical SAM Enzyme PqqE. Biochemistry.

[43] Aono S., Bryant F. O., Adams M. W. (1989). A Novel and Remarkably Thermostable
Ferredoxin from the Hyperthermophilic Archaebacterium Pyrococcus Furiosus. J. Bacteriol..

[44] Park J. B., Fan C. L., Hoffman B. M., Adams M. W. (1991). Potentiometric and
Electron Nuclear Double Resonance Properties of the Two Spin Forms
of the [4Fe-4S]+ Cluster in the Novel Ferredoxin from the Hyperthermophilic
Archaebacterium Pyrococcus Furiosus. J. Biol.
Chem..

[45] Brereton P. S., Duderstadt R. E., Staples C. R., Johnson M. K., Adams M. W. W. (1999). Effect
of Serinate Ligation at Each of the Iron Sites of the [Fe4S4] Cluster
of Pyrococcus Furiosus Ferredoxin on the Redox, Spectroscopic, and
Biological Properties. Biochemistry.

[46] Wang W., Li J., Wang K., Huang C., Zhang Y., Oldfield E. (2010). Organometallic
Mechanism of Action and Inhibition of the 4Fe-4S Isoprenoid Biosynthesis
Protein GcpE (IspG). Proc. Natl. Acad. Sci.
U. S. A..

[47] Skeel B. A., Suess D. L. M. (2023). Exploiting Molecular Symmetry to Quantitatively Map
the Excited-State Landscape of Iron-Sulfur Clusters. J. Am. Chem. Soc..

[48] Niu S., Ichiye T. (2009). Cleavage of [4Fe—4S]-Type
Clusters: Breaking
the Symmetry. J. Phys. Chem. A.

[49] Skeel B. A., Suess D. L. M. (2025). Iron-Sulfur Clusters:
The Road to Room Temperature. JBIC J. Biol.
Inorg. Chem..

[50] Boll M., Fuchs G., Tilley G., Armstrong F. A., Lowe D. J. (2000). Unusual Spectroscopic and Electrochemical
Properties
of the 2­[4Fe-4S] Ferredoxin of Thauera Aromatica. Biochemistry.

[51] Moore W. M., Spence J. T., Raymond F. A., Colson S. D. (1963). Photochemistry of
Riboflavin. I. The Hydrogen Transfer Process in the Anaerobic Photobleaching
of Flavins. J. Am. Chem. Soc..

[52] Radda G. K., Calvin M. (1964). Chemical and Photochemical Reductions of Flavin Nucleotides
and Analogs*. Biochemistry.

[53] Penzer G. R., Radda G. K. (1968). The Chemistry of
Flavines and Flavoproteins. Photoreduction
of Flavines by Amino Acids. Biochem. J..

[54] Grady J. K., Chasteen N. D., Harris D. C. (1988). Radicals
from “Good’s”
Buffers. Anal. Biochem..

[55] Kirsch M., Lomonosova E. E., Korth H.-G., Sustmann R., de Groot H. (1998). Hydrogen Peroxide
Formation by Reaction of Peroxynitrite with HEPES and Related Tertiary
Amines: IMPLICATIONS FOR A GENERAL MECHANISM*. J. Biol. Chem..

[56] Hausladen D. M., Peña J. (2023). Organic Buffers
Act as Reductants of Abiotic and Biogenic
Manganese Oxides. Sci. Rep..

[57] Artz J. H., Mulder D. W., Ratzloff M. W., Lubner C. E., Zadvornyy O. A., LeVan A. X., Williams S. G., Adams M. W. W., Jones A. K., King P. W., Peters J. W. (2017). Reduction
Potentials of [FeFe]-Hydrogenase
Accessory Iron-Sulfur Clusters Provide Insights into the Energetics
of Proton Reduction Catalysis. J. Am. Chem.
Soc..

[58] Bertrand P. (1985). Electron Transfer
between Biological Molecules Coupled by an Exchange Interaction. Chem. Phys. Lett..

[59] Bertrand P. (1986). Some Important
Concepts in the Current Theories of Electron Transfer in Biological
Systems. Biochimie.

[60] Bertrand, P. Application of Electron Transfer Theories to Biological Systems. In Long-range electron transfer in biology; Springer, 1991; pp 1–47.

[61] Heelis P.
F., Phillips G. O. (1979). Photoreduction
Reactions of Flavin Coenzymes - A Laser
Flash Photolysis Study. Photobiochem. Photobiophys..

[62] Penzer, G. R. ; Radda, G. K. [152] Photochemistry of Flavins. In Methods in Enzymology; Academic Press, 1971; Vol. 18, pp 479–495. 10.1016/S0076-6879(71)18109-8.

[63] Heelis P. F. (1982). The Photophysical
and Photochemical Properties of Flavins (Isoalloxazines). Chem. Soc. Rev..

[64] Zeldes H., Livingston R. (1977). Electron Spin
Resonance Study of Radicals Produced
by One-Electron Reduction of Nicotinic Acid, Nicotinamide, and Methyl
Nicotinate. J. Magn. Reson. 1969.

[65] Farrington J. A., Land E. J., Swallow A. J. (1980). The One-Electron
Reduction Potentials
of NAD. Biochim. Biophys. Acta BBA - Bioenerg..

[66] Jacoby
Morris K., Barnard D. T., Narayanan M., Byrne M. C., McBride R. A., Singh V. R., Stanley R. J. (2022). Comparing
Ultrafast Excited State Quenching of Flavin 1,N6-Ethenoadenine Dinucleotide
and Flavin Adenine Dinucleotide by Optical Spectroscopy and DFT Calculations. Photochem. Photobiol. Sci..

[67] Elliott J. I., Brewer J. M. (1978). The Inactivation
of Yeast Enolase by 2,3-Butanedione. Arch. Biochem.
Biophys..

[68] Wiley S., Griffith C., Eckert P., Mueller A. P., Nogle R., Simpson S. D., Köpke M., Can M., Sarangi R., Kubarych K., Ragsdale S. W. (2024). An Alcove at the
Acetyl-CoA Synthase
Nickel Active Site Is Required for Productive Substrate CO Binding
and Anaerobic Carbon Fixation. J. Biol. Chem..

[69] Fish, W. W. [27] Rapid Colorimetric Micromethod for the Quantitation of Complexed Iron in Biological Samples. In Methods in Enzymology; Academic Press, 1988; Vol. 158, pp 357–364. 10.1016/0076-6879(88)58067-9. 3374387

[70] Riemer J., Hoepken H. H., Czerwinska H., Robinson S. R., Dringen R. (2004). Colorimetric
Ferrozine-Based Assay for the Quantitation of Iron in Cultured Cells. Anal. Biochem..

[71] Aliverti A., Curti B., Vanoni M. A. (1999). Identifying
and Quantitating FAD
and FMN in Simple and in Iron-Sulfur-Containing Flavoproteins. Flavoprotein Protoc..

[72] Bergmeyer, H. U. Methods of Enzymatic Analysis, 2nd ed.; Academic Press: New York, 1974; Vol. 1.

[73] Siegel J. M., Montgomery G. A., Bock R. M. (1959). Ultraviolet Absorption Spectra of
DPN and Analogs of DPN. Arch. Biochem. Biophys..

[74] Fruscione F., Sturla L., Duncan G., Van Etten J. L., Valbuzzi P., De Flora A., Di Zanni E., Tonetti M. (2008). Differential
Role of NADP+ and NADPH in the Activity and Structure of GDP-D-Mannose
4,6-Dehydratase from Two Chlorella Viruses *. J. Biol. Chem..

[75] Stoll S., Schweiger A. (2006). EasySpin, a Comprehensive Software Package for Spectral
Simulation and Analysis in EPR. J. Magn. Reson..

[76] Stoll, S. Chapter Six - CW-EPR Spectral Simulations: Solid State. In Methods in Enzymology; Qin, P. Z. , Warncke, K. , Eds.; Academic Press, 2015; Vol. 563, pp 121–142. 10.1016/bs.mie.2015.06.003. 26478484

