# Supplementary material for: Photoinduced Electron Transfer Informs on Pathway Coupling in Flavin-Based Electron Bifurcation
Source: ACS Bio Med Chem Au. 2026 Feb 6;6(1):78–89. doi: 10.1021/acsbiomedchemau.5c00232 (PMC12921514; doi:10.1021/acsbiomedchemau.5c00232)
Supplement: Supplementary file 1 [file bg5c00232_si_001.pdf]

Supporting Information

for

Photoinduced Electron Transfer Informs on Pathway Coupling in Flavin-Based Electron Bifurcation.

Seth A. Wiley, Carolyn E. Lubner*

Biosciences Center, National Laboratory of the Rockies, Golden, CO, USA

Table of Contents

Table S1 3

Supporting Figures:..5

Figure S1: 5

Table S2: 5

Figure S2: 6

Figure S3: 7

Table S3: 7

Figure S4: 8

Figure S5: 9

Figure S6: 10

Figure S7: 11

Figure S8 12

Table S4: 13

Figure S9: 14

Figure S10: 15

Figure S11 16

Figure S12: 17

Figure S13: 18

Figure S14: 19

Figure S15 20

Figure S16: 21

Table S5: 21

Figure S17: 22

Figure S18: 23

Figure S19: 24

Figure S20: 25

Figure S21: 26

Figure S22: 27

Supporting References: 28

Table S1: EPR Sample Conditions used in this study.

Sample	Concentration (µM)	†Measured [Nfn] (µM)	Condition	Experiment	Notes
$NfnL_{PE\text{-}I}$	140	124 ± 1	8.8 mM NADPH	Initial Photoexcitation	Pre- vs Post-illum
$NfnL_{PE\text{-}T}$	140	121 ± 4	8.8 mM NADPH	Phototitrations/Max Photoexcitation	Largest signals
$NfnL_{Red}$	100	76 ± 2	10x DTH	Reduced NfnL ref.	Reduced ref.
$NfnL_{Dist}$	100	76 ± 2	1x DTH	NfnL -Distal ref.	Distal cluster ref.
$NfnL_{Ox}$	150	147 ± 2	As-Isolated	Photocontrol	Minor (~1%) signals
FAD	200	-0.6 ± 0.3	Oxidized	Photocontrol	No signal
NADPH	8800	-1.3 ± 0.7	Reduced	Photocontrol	No signal
$NfnSL_{Ox}$	150	135 ± 4	As-Isolated	NSQ ref.	Linewidth ref.

†Concentrations of Nfn were assessed by the rose bengal method[1–3] in replicates of $n \geq 3$.

Power Saturation of EPR Spectra

Using the following equation according to Galli *et al.*, electron paramagnetic resonance (EPR) power saturation curves (**Figures S1B** and **S3B**) can be fit to the following equation to pull out the saturation half-power, or $P_{1/2}$ parameter as a means of identifying relaxation characteristics for different paramagnetic species, either alone or in a mixed population[4–6]:

$$\log\left(\frac{S}{\sqrt{P}}\right) = \log(a) - 0.5 * b * \log\left(1 + \left(\frac{x}{P_{\frac{1}{2}}}\right)\right)$$ **Equation S1**[4]

The overall signal, $\log\left(\frac{S}{\sqrt{P}}\right)$, located on the *y-axis* and represented in **Figures S1B** and **S3B**, will appear flat if the signals are not saturating. When the species' relaxation begins to saturate as a function of power, the *y-axis* value will begin to exponentially reduce, appearing hyperbolic. Using **Equation S1**, we can better understand the impact of mixed species on a particular *g*-value of interest, such as those representative of the proximal cluster (*g* = 1.972, 1.954, 1.924) and the distal cluster (*g* = 2.034, 1.934, 1.896). In our case, we used these relaxation characteristics to determine if the previously assigned[7] *g*-values for the proximal cluster were accurate for the *g* = 2.035, which proved to have nearly no major influence from the proximal cluster as observed at *g* = 1.974 (**Figures S1** and **S3**, and **Tables S2** and **S3**).

Calculation of Fraction of Reduced L-FAD after NADPH Treatment at Equilibrium

In order to estimate the equilibrium ratio ("*Q*") of reduced L-FAD after NADPH treatment based on standard potentials at room temperature of the L-FAD[8] (midpoint = -435 mV) and NADPH[9] (fully reduced ≈ -380 mV), we can use the Nernst equation to find this ratio assuming the following:

$$Q = \frac{[LFAD_{red}][NADP^{+}]}{[LFAD_{ox}][NADPH]} \simeq \frac{[LFAD_{red}]}{[LFAD_{ox}]}$$ **Equation S2**

$$\Delta E^{\circ} = E^{\circ}_{LFAD} - E^{\circ}_{NADPH} = -435\ mV - -380\ mV = -55\ mV = -0.055\ V$$ **Equation S3**

Taking the Nernst equation at equilibrium:

$$0 = \Delta E^{\circ} - \left(\frac{RT}{nF}\right) * \ln(Q)$$ **Equation S4**

After rearranging the equation and inserting known values we get:

$$\ln(Q) = \frac{\Delta E^{\circ}}{\left(\frac{R*298.15\ K}{nF}\right)} = \frac{-0.055\ V*n}{0.0257} = -4.2801$$ **Equation S5**

Correcting for natural logarithms:

$$e^{(\ln(Q))} = Q = e^{-4.2801} = 0.01384 = 1.38\%\ reduced\ NfnL\ FAD$$ **Equation S6**

This value for the reduced L-FAD is consistent with small percentage observed experimentally by UV-Visible spectroscopy, as depicted in **Figure S12**.

Supporting Figures:

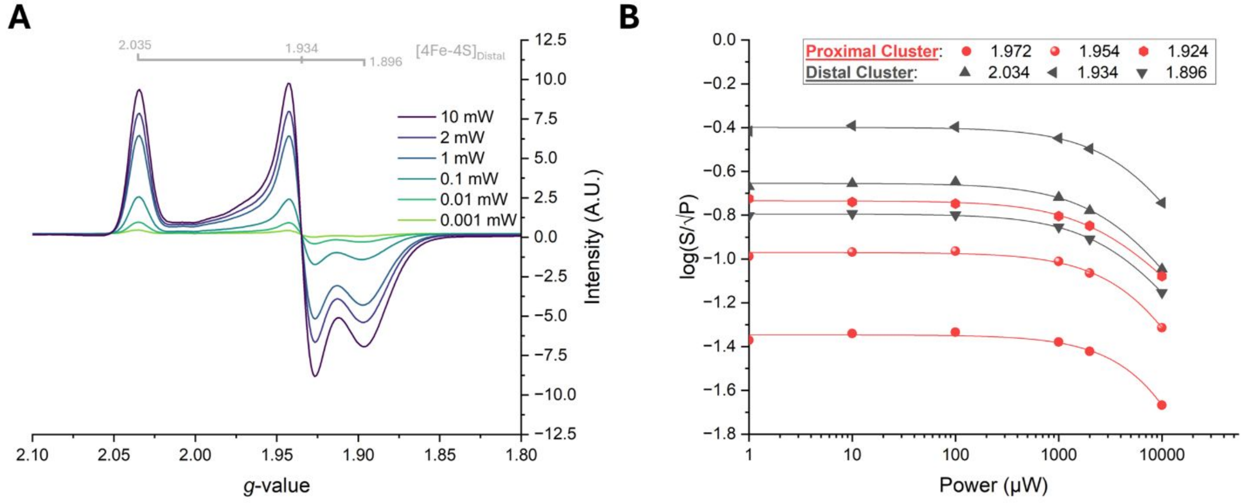

Figure S1: EPR power dependence of the 1x DTH reduced NfnL sample, showing the saturation behavior of the distal [4Fe-4S] cluster signal. Panel **A** depicts the power dependence of the 1x DTH sample at 20 K, with the signal's dependence on power for the distal [4Fe-4S] cluster. Panel **B** depicts the well-behaved saturation behavior of characteristic *g*-values for the proximal (*red*) and distal (*charcoal*) [4Fe-4S] clusters, clearly indicating only the distal cluster is present in this condition. Power saturation fit values corresponding to the data in panel **B** are located in **Table S2**. Spectra were taken at 20 K, with modulation amplitude of 10 G, and powers listed.

Table S2: Fitting parameters for the saturation behavior of the selected g-values for the 1x DTH reduced NfnL shown above in **Figure S1B**. Since there is only one species present (distal cluster) in this condition, the power saturation fits are well-behaved and consistent.

Proximal Cluster								
g-value	a	a_{err}	b	b_{err}	$P_{1/2}$	$P_{1/2,err}$	χ^2	R^2
1.972	0.045	0.001_0	6.0	$14._6$	34000	99000	3.1e-4	0.988
1.954	0.107	0.001_8	2.4	1.4_6	11000	8900	1.6e-4	0.995
1.924	0.184	0.001_9	1.1	0.1_6	3300	800	5.9e-5	0.998
Distal Cluster								
g-value	a	a_{err}	b	b_{err}	$P_{1/2}$	$P_{1/2,err}$	χ^2	R^2
2.034	0.221	0.003_6	1.6	0.4_0	4800	2000	1.4e-4	0.996
1.934	0.398	0.006_3	1.9	0.7_8	7700	4700	1.4e-4	0.995
1.896	0.160	0.001_1	1.4	0.1_7	4600	900	2.7e-5	0.999

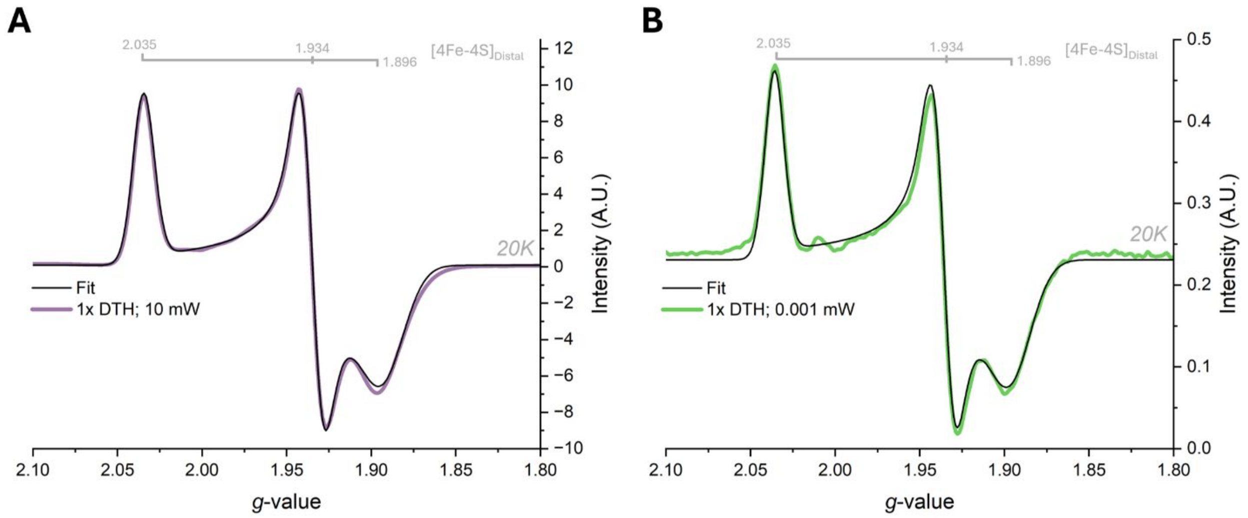

<u>Figure S2:</u> Simulations of 1x DTH EPR spectra at the highest (10 mW; *left*, **A**) and lowest (0.001 mW; *right*, **B**) powers probed in this study. No significant differences in distal cluster fits were observed despite orders of magnitude in power, indicating the fits are consistent over large power differences.

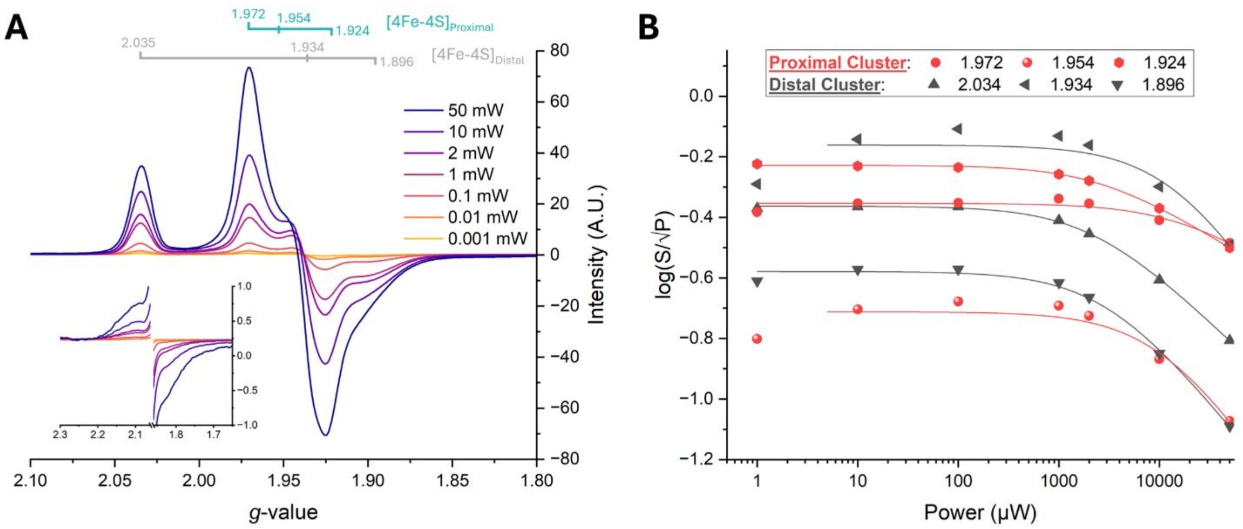

Figure S3: EPR power dependence of the 10x DTH reduced NfnL sample, showing both proximal and distal [4Fe-4S] cluster signals. Panel **A** depicts the power dependence of the 10x DTH sample at 20 K, showing proximal [4Fe-4S] cluster signal saturates at a higher power relative to the distal [4Fe-4S] cluster (**Figure S1**). Inset graph in panel **A** shows additional intensity indicative of cluster-cluster coupling flanking the main g ~ 2 region. Panel **B** depicts the saturation behavior of characteristic g-values for the proximal (*red*) and distal (*charcoal*) [4Fe-4S] clusters, clearly indicating different saturation behavior between the two species despite spectral overlap. Power saturation fit values corresponding to the data in panel **B** are located in **Table S3**. Spectra were taken at 20 K, with modulation amplitude of 10 G, and powers listed.

Table S3: Fitting parameters for the saturation behavior of the selected g-values for the 10x DTH reduced NfnL shown above in **Figure S3B**. Due to the presence of multiple overlapping g-values, the power saturation fits are convoluted. Representative g-values for each species are bolded, showing the ability to resolve the independent species. The fact that the **b** value for most species is less than 1 indicates these values are coupling with one another and influencing each other's overall relaxation characteristics.

Proximal Cluster								
**g-value**	**a**	**a_{err}**	**b**	**b_{err}**	**$P_{1/2}$**	**$P_{1/2,err}$**	**χ^2**	**R^2**
1.972	0.44	0.01_0	0.4	0.4_0	15000	27000	3.8e-4	0.904
1.954	0.19	0.01_2	1.0	0.8_4	11000	19000	0.0029	0.902
1.924	0.591	0.002_4	0.42	0.01_6	2700	300	9.7e-6	0.999
Distal Cluster								
**g-value**	**a**	**a_{err}**	**b**	**b_{err}**	**$P_{1/2}$**	**$P_{1/2,err}$**	**χ^2**	**R^2**
2.034	0.433	0.003_1	0.65	0.02_5	2300	300	2.9e-5	0.999
1.934	0.69	0.06_1	1.1	$1._6$	15000	44000	0.0058	0.796
1.896	0.264	0.006_7	0.8	0.1_1	3200	1200	3.9e-4	0.993

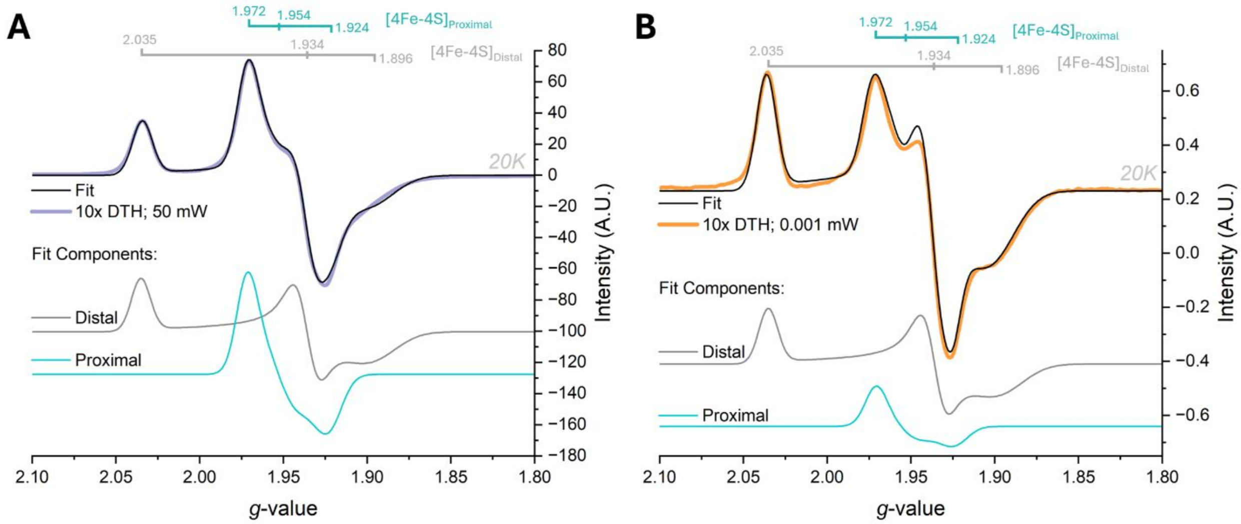

Figure S4: Simulations of 10x DTH EPR spectra at the highest (50 mW; *left*, **A**) and lowest (0.001 mW; *right*, **B**) powers probed in this study. While proximal (cyan) component magnitudes were different, overall *g*-value fits were consistent amongst the two powers, indicating the newly reassigned semi-axial spectrum shape is more representative of the proximal [4Fe-4S] cluster lineshape.

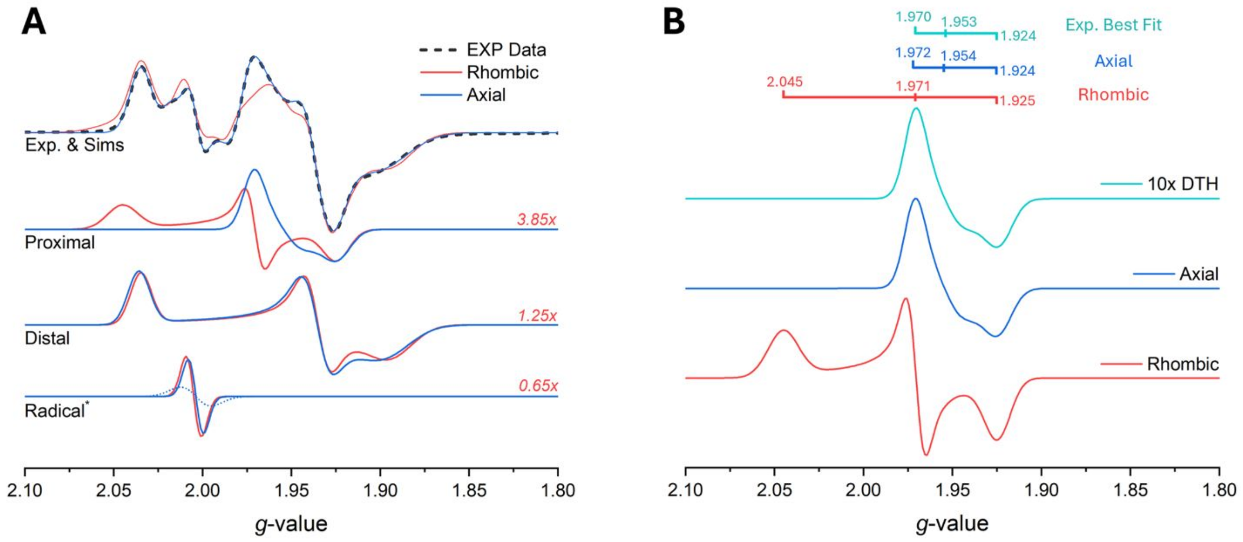

<u>Figure S5:</u> Easyspin simulations indicate semi-axial shape for the proximal [4Fe-4S] cluster by deconvoluting the multicomponent spectra observed in this report. Panel **A** depicts the 4.5 hour illuminated experimental data at 20 K (*black* dotted line), the full simulated traces (*red* and *blue* solid lines), and the subsequent components (proximal, distal, and radical) for each fitting condition. The newly reassigned semi-axial ("Axial"; *blue*; RMSD = 0.1046) *g*-values are compared to the previously assigned rhombic *g*-values ("Rhombic"; *red*; RMSD = 1.0294). While the distal cluster and radical components have nearly identical *g*-values for both shape assignment conditions, the overall fit is better when using semi-axial *g*-values for the proximal cluster. The Radical* section for the illumination spectrum depicts 2 components: one with the clear ASQ radical, and the other likely representing the coupling signal explained further in the proceeding Figure **S6A**. Panel **B** directly compares the fits for the proximal [4Fe-4S] cluster component between the 10x DTH reference (*cyan*), the newly refined semi-axial fit (*blue*), and the previously assigned rhombic fit (*red*), with their associated *g*-values labeled above.

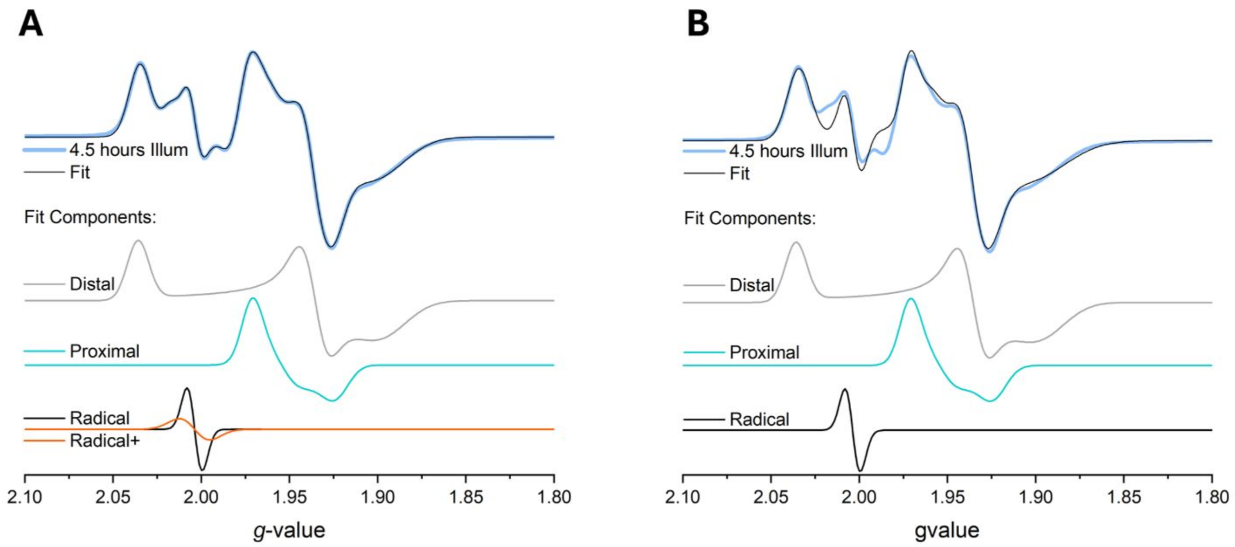

<u>Figure S6:</u> Illumination data fits best to a 4-component system at 20 K. Panel **A** (*left*) shows the 4.5 hour illumination spectrum fit to 4 components (RMSD = 0.1046), with panel **B** (*right*) fit to 3 components (RMSD = 0.3847). The overall fit quality is decent when the spectrum is fit to 3 components but does not represent the *g* ~ 2.00 region well, altering the quality of the overall fit. While the exact identity of the 4th component ("Radical+") is not specifically known, the other 3 known components fit exceptionally well when the additional signal is accounted for. Since the 4th component is unobserved above 20 K (**Figure 2A**) it is unlikely to be an additional radical species and may indicate the triplet state coupling ($S_{Tot} = S_{Rad} + S_{Prox} = ½ + ½ = 1$) between the ASQ and proximal [4Fe-4S] cluster.[10–12]

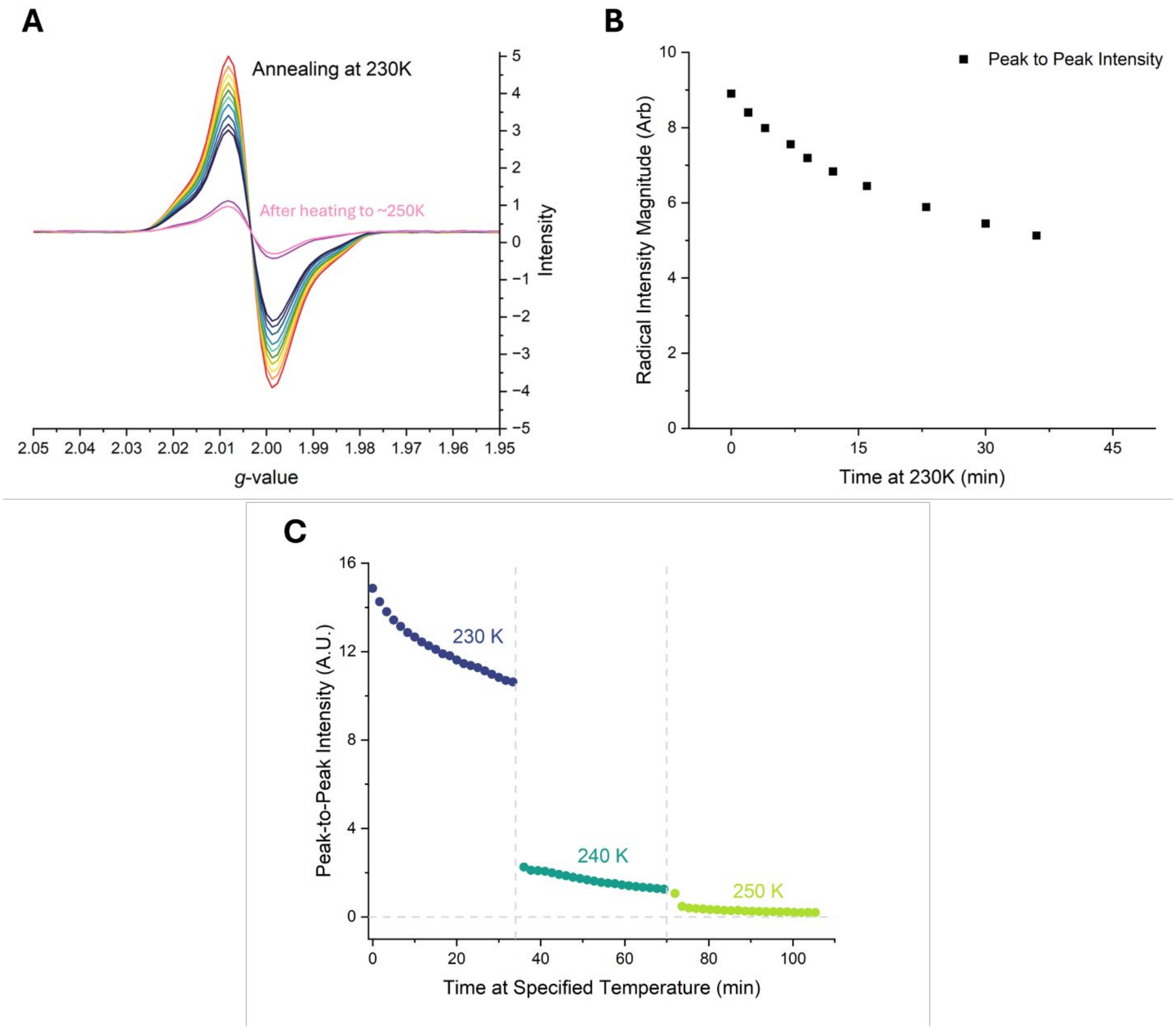

<u>Figure S7:</u> Stability of the ASQ radical at 230 to 250 K. Panel **A** shows the decay in the emergent radical species spectrum over the course of 45 minutes at 230 K and the increased signal decay at 250 K. Panel **B** shows the peak-to-peak decay in the radical intensity at 230 K, showing that the radical has an overall long-lived lifetime, about 35 min. Panel **C** shows a significant increase in decay between 230 and 240 K with the sample returning to near zero at 250 K. Panel **A** and **B** data are from the initial illumination sample (**Figure 2**), and panel **C** data comes from the 4.5 hr illumination.

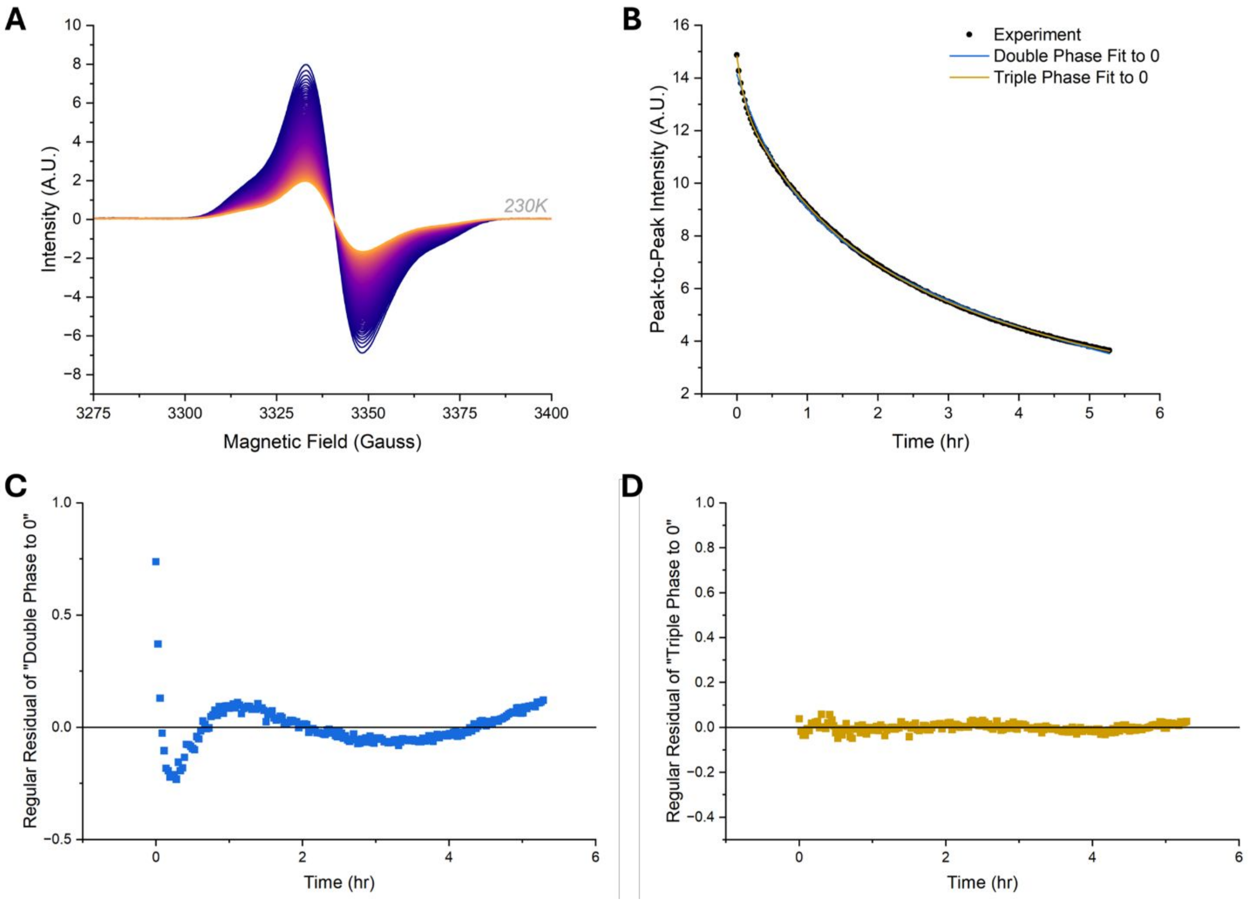

Figure S8: Stability of the ASQ radical at 230 K over the course of 5 hours. Panel **A** depicts the spectral decay corresponding to ASQ recombination at 230 K, slowly returning to the initial condition (*violet* to *yellow*) after a period of 5 hours, dropping from a peak-to-peak intensity of ~ 15 A.U. to ~ 4. Even after 5 hours, the remaining peak-to-peak intensity is ~ 4 A.U., roughly 26% of the starting intensity. Panel **B** shows the decay curve of the peak-to-peak intensity at 230 K observed in panel **A**. Fit parameters are located in the proceeding **Table S4**, with the best fit condition for the signal decay back to initial is shown in *yellow*, with a triple phase function of 0.1020 ± 0.002 hours (11.1% intensity), 1.23 ± 0.02 hours (35.1% intensity), and 6.53 ± 0.08 hours (53.8% intensity) (R^2 = 0.99996; $\chi^2 = 3.03 * 10^{-4}$). Panels **C** and **D** both depict the residuals for the two best fits, showing the *blue* double phase fit (**C**) is indeed missing a minor but important component when compared to the *yellow* triple phase fit (**D**). Why this decay is dependent on a triple phase fit is worth noting and may reflect three states with differing readiness for electron recombination. These 3 phases would most likely represent: 1) an electron in the proximal [4Fe4S] ready for transfer to the ASQ; 2) an electron in the distal [4Fe4S] cluster that must transfer to the proximal cluster before recombination; and 3) an additional spectroscopically silent "gated" mechanism only seen in biochemical experiments.[13]

Table S4: Exponential fitting parameters from **Equation S7** used for the ASQ recombination experiment at 230 K shown in **Figure S8**. The final peak-to-peak intensity (y0) for the decay of the radical was locked to zero since as the temperature is raised above 230 K, the radical should decay completely back to the baseline, as seen in the spectra before illumination and after annealing at higher temperatures (see **Figures 2** and **5**, *Dark*, *Anneal*, and *1x DTH* traces).

Fitting Parameter	Double Phase Fit (*blue* trace; S8C)	Triple Phase Fit (*yellow* trace; S8D)
y0†	0 ± 0	0 ± 0
A1 (intensity) (%)	4.22 ± 0.09 (29.9%)	1.64 ± 0.02 (11.1%)
τ_1 (hours)	0.65 ± 0.02	0.102 ± 0.003
A2 (intensity) (%)	9.91 ± 0.09 (70.1%)	5.21 ± 0.07 (35.1%)
τ_2 (hours)	5.11 ± 0.07	1.23 ± 0.02
A3 (intensity) (%)	-	7.98 ± 0.08 (53.8%)
τ_3 (hours)	-	6.53 ± 0.08
χ^2	$90.2 \bullet 10^{-4}$	$3.0 \bullet 10^{-4}$
R^2	0.99874	0.99996

†Locked to "0" as radical intensity has been observed to decay back to the initial condition.

$$\boldsymbol{Intensity = y0 + A1 * e^{\left(-\frac{x}{\tau_1}\right)} + A2 * e^{\left(-\frac{x}{\tau_2}\right)}} + A3 * e^{\left(-\frac{x}{\tau_3}\right)} \qquad \textbf{Equation S7}$$

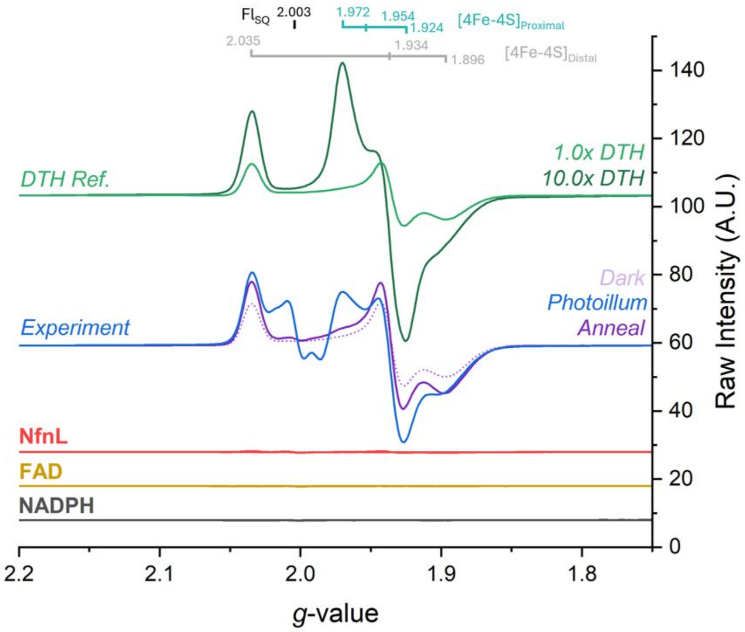

Figure S9: Raw photocontrol intensities (*red*, *gold*, *charcoal*) in comparison to initial illumination experiment (*blues*) and 1x and 10x DTH references (*green*). Maximum observed intensity of photocontrol experiments was no more than 1% of the total signal observed in the photogenerated and DTH reduced experiments. All spectra shown are at 20 K, taken with 10 mW power and 10 G modulation amplitude.

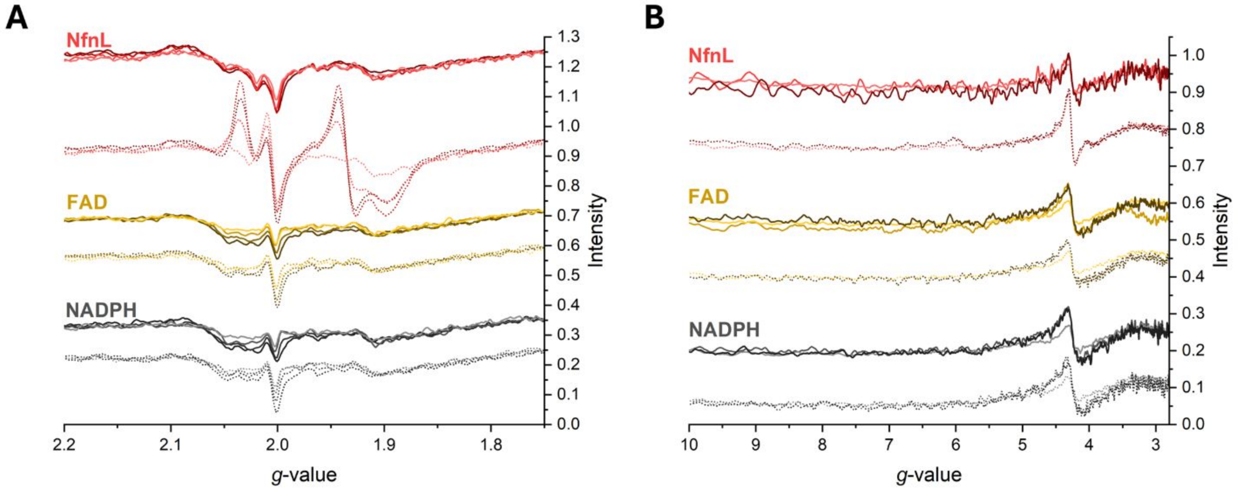

<u>Figure S10:</u> Magnified EPR spectra of photocontrol experiments. Solid lines (—) were spectra before illumination, and dotted lines (···) were taken after illumination at 405 nm for 1 hour. No significant signals are observed prior to illumination for any sample for either the *g* ~ 2 (panel **A**) or low-field (panel **B**) regions. Of the controls, only NfnL alone (*red*) has a noticeable photoinduced signal, which shows a minor amount of distal cluster and a radical signal in the *g* ~ 2 region, and interestingly, increased intensity on top of the "junk iron" signal at *g* = 4.3 and a small feature around *g* ~ 6 unique to the illuminated NfnL alone. Aside from the photoinduced NfnL signal, the predominant spectral features around the *g* ~ 2 region are contributed by the cavity itself, which are seen in the FAD (*gold*) and NADPH (*charcoal*) controls. Spectra in panel **A** were taken at 20, 30, 50 and 80 K (darker to lighter traces) with 10 mW power and 10 G modulation amplitude. Spectra in panel **B** were taken at 3.6, 4, and 20 K (dark to lighter traces) with 10 mW power and 10 G modulation amplitude.

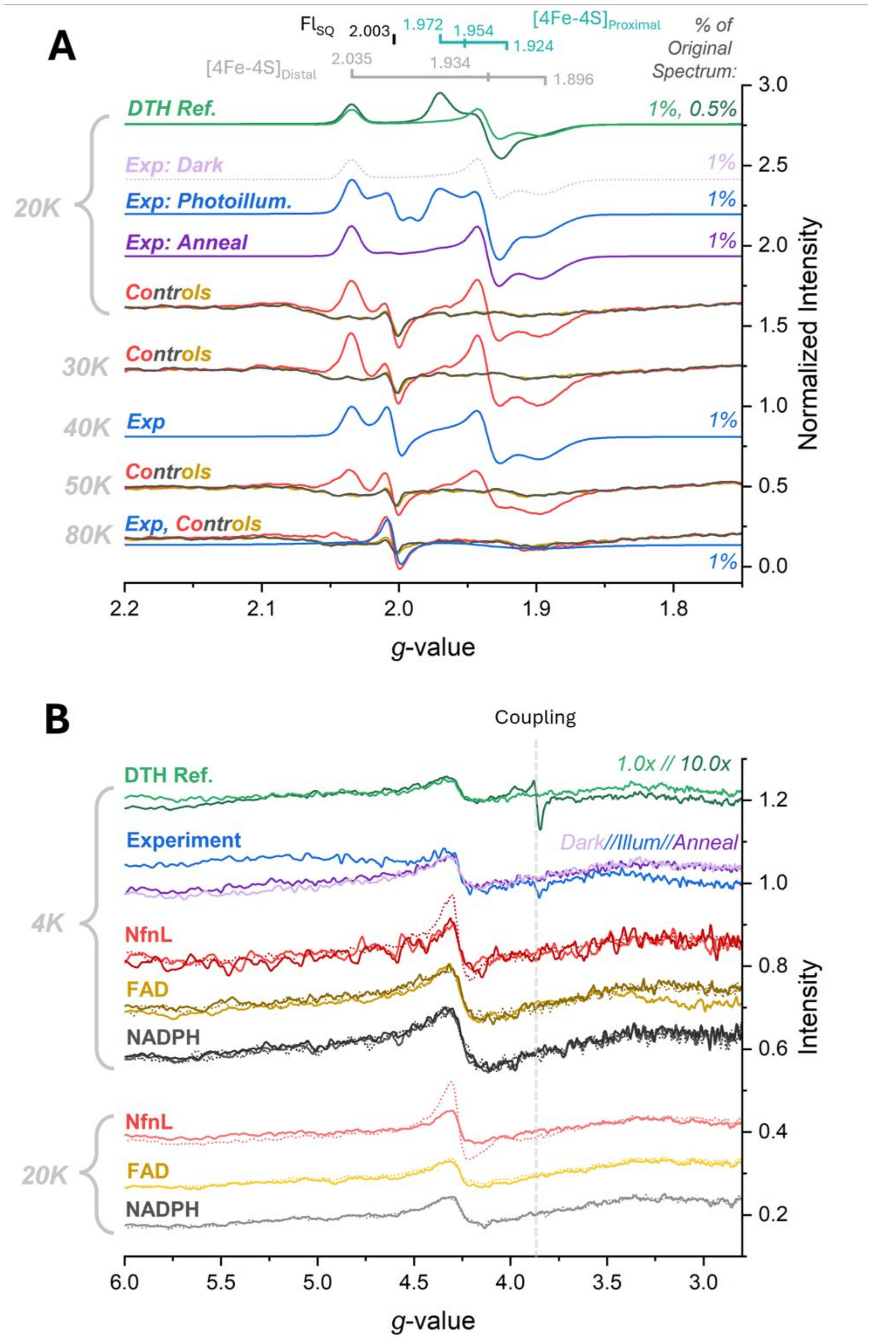

Figure S11: Intensity-normalized photocontrol EPR data comparing spectral identities to the illumination data observed in **Figure 2**. Panel **A** depicts *g* ~ 2 region data by temperature for illumination experiments (*blue*, *purple* spectra) compared to DTH references (*green* spectra) and photocontrols (*red*, *gold*, *charcoal*). Main illuminated NfnL (*red*) spectral features align with the distal cluster signal and radical feature, implying some minor electron transfer (~1% of that in **Figure 2**) is occurring, either from solution or internally in NfnL as stated in **Figure S10A**. Panel **B** shows all aforementioned samples but for the low-field region, and reiterates that a signal appears on top of *g* ~ 4.3 for the NfnL (*red*) sample after illumination previously stated in **Figure S10B**.

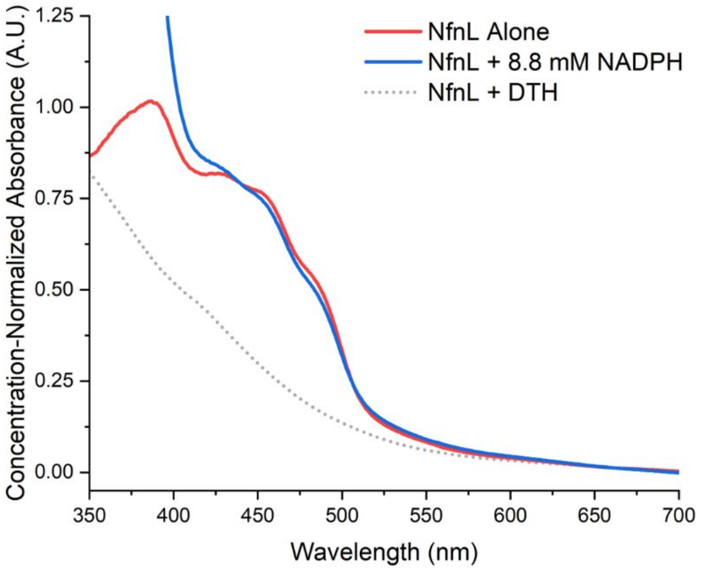

<u>Figure S12:</u> NADPH treatment does not appreciably reduce the L-FAD in NfnL. Comparing the UV-Visible spectra between NfnL alone (*red*, solid line), NADPH-treated NfnL (*blue*, solid line), and a completely reduced dithionite-treated NfnL (*grey*, dotted line), only marginal reduction (≤ 5%) of the oxidized L-FAD was observed for the NADPH treatment, suggesting the L-FAD must be in the oxidized state for photoexcitation rather than in any other redox state, particularly opposite the reduced HQ state. These UV-vis data are consistent with a calculated ratio of L-FAD reduction of ~1.4% using the Nernst equation under equilibrium conditions (*vida supra*; **Equations S2-S6**), where the redox potential used for NADPH was ~ -380 mV[9] and the midpoint potential of the L-FAD_{OX}/L-FAD_{HQ} couple has been shown to be -435 mV.[8] The spectra were normalized to their concentration as outlined in **Table S1**.

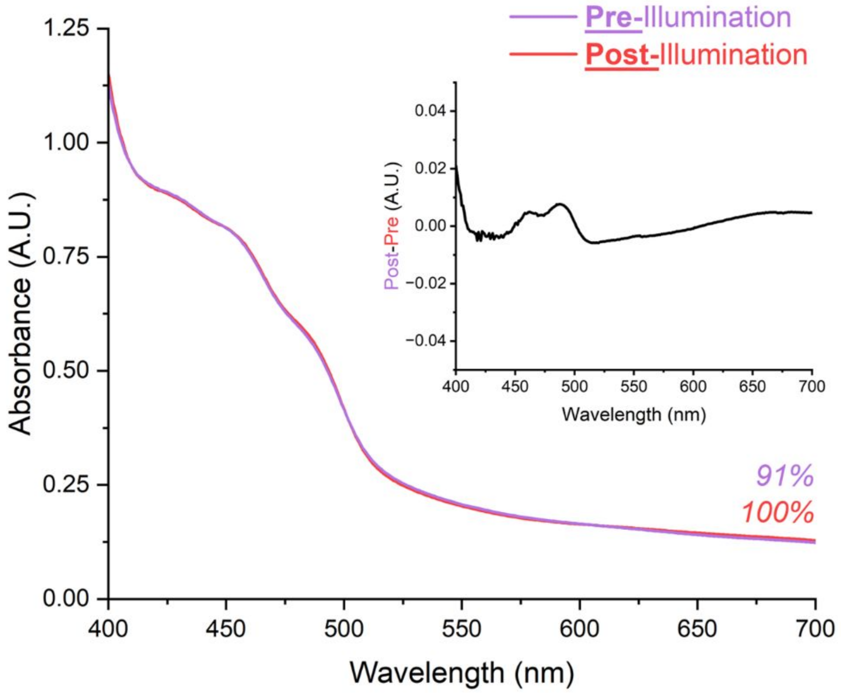

Figure S13: Normalized pre-illumination (*lavender*) and post-illumination (*red*) UV-Visible spectra of the representative phototreated EPR spectra presented in **Figure 2**. Pre- and post-illumination spectra overlay nearly identically, but the preillumination spectrum was scaled to 91% (within margin of error for colorimetric concentration validation) in order to compare to the post-illumination spectrum. The lack of any additional spectral features or changes confirms no photodamage was caused under the illumination experimental conditions. The inset shows the difference (post minus pre) spectrum after illumination, reiterating no photodamage has occurred, and that the spectral changes are likely due to some minor oxidation (less than 5%) to the NfnL L-FAD.

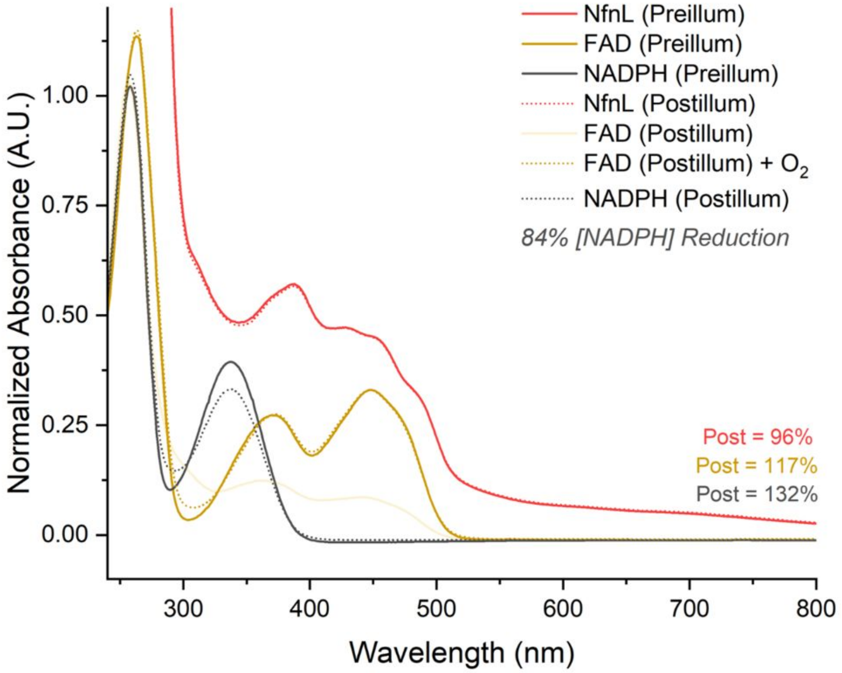

<u>Figure S14:</u> Normalized UV-Visible spectral comparisons of pre- and post-illumination photocontrol samples, showing photodegradation is not a significant contributor to the EPR results. Interestingly, upon 1 hour of illumination under identical conditions, the FAD control appeared to photoreduce to the HQ state (*pale yellow* spectrum; **Figure S15**), and the electron source is hypothesized to be from the HEPES buffer solution itself.[14–16] Upon oxidation by ambient O_2 (5 minutes), the FAD returned to the initial spectrum before illumination, confirming the intermediate was photoreduced rather than photodegraded. This FAD photoreduction is unlikely to be significant relative to the data in **Figure 2**, considering any of NfnL's L-FAD is bound internally and considerably less available to solvent-sourced electrons, which is validated by the lack of any significant EPR signals (**Figure S9-11**) or L-FAD reduction observed via UV-Vis in NfnL alone (*red* traces above). Normalized intensity differences between the pre- and post-illumination spectra are reflected in the bottom right corner as percentages, and only NADPH (*charcoal* traces) is greater than 20% different, likely due to some minor photooxidation to $NADP^+$, as observed at 340 nm.

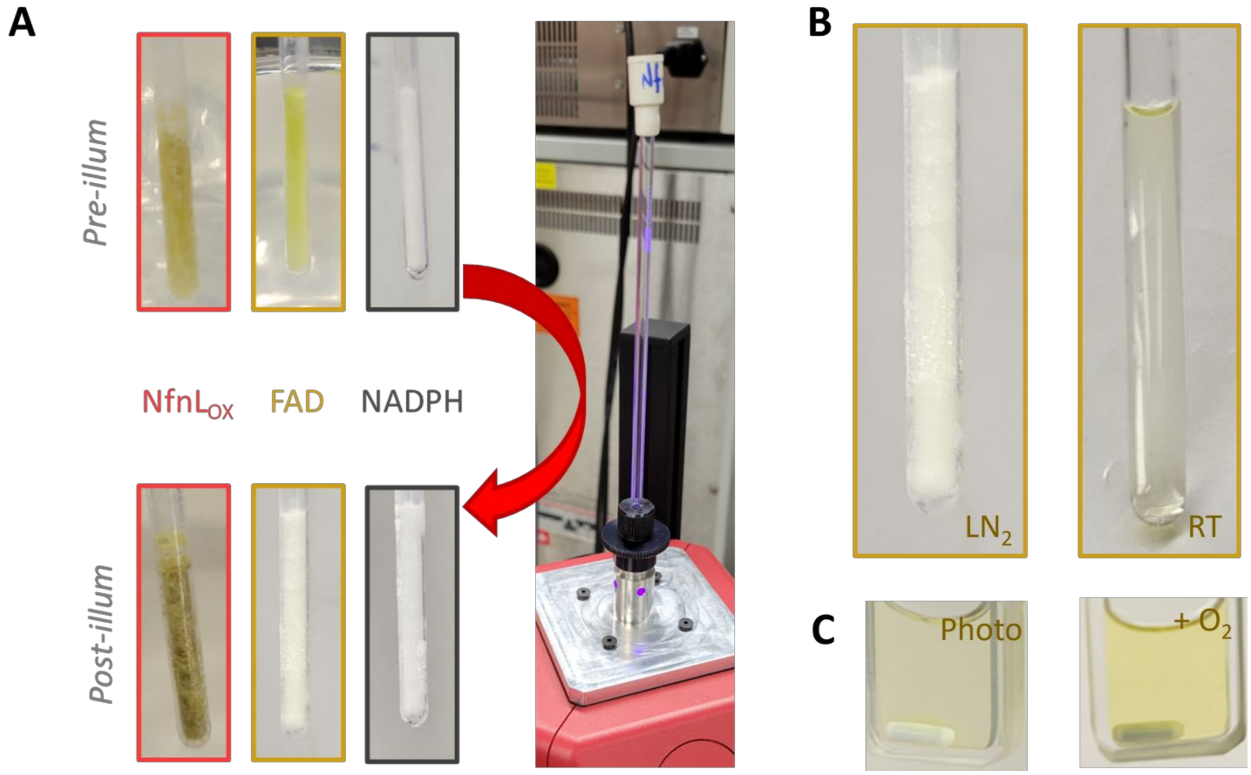

Figure S15: Images of pre- and post-illumination photo controls shown in **Figures S9-S11 & S14**. Subfigure **A** shows that some visible changes were evident to the naked eye after illumination for the photocontrol experiments, where NfnL alone appeared a bit darker in hue and the FAD control appeared significantly paler. Subfigure **B** shows the FAD control's color change remained upon returning the sample to room temperature (RT), implying that the changes in the color are stable and from photoreduction rather than from an unstable and transient intermediate. Subfigure **C** shows the return in yellow color of the illuminated FAD sample when exposed to ambient O_2, as depicted in the UV-Vis spectra (**Figure S14**), implying the paler change was from the reduction of FAD to $FADH_2$ rather than photodamage.

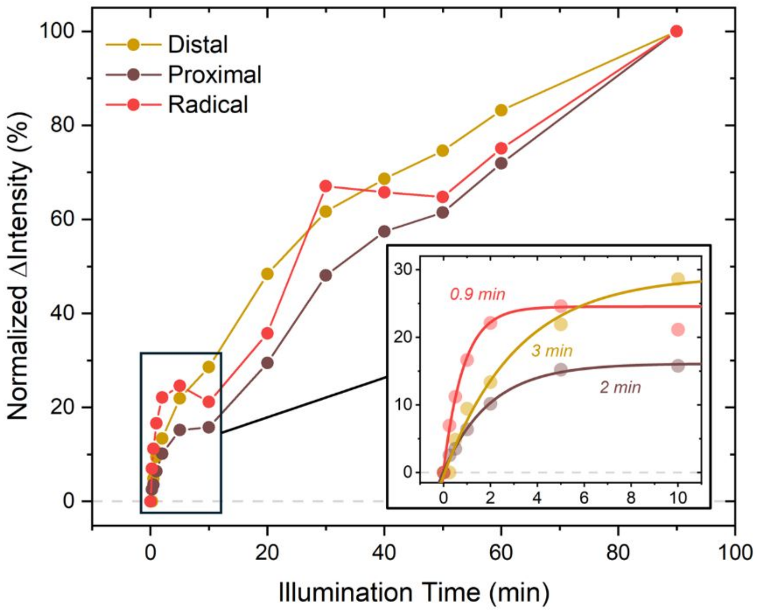

Figure S16: Radical generation precedes cluster reduction. Over the course of the 90 minute phototitration experiment (**Figure 4**), all paramagnetic species (radical at *g* = 2.003; distal [4Fe-4S] cluster at *g* = 2.035; and proximal [4Fe-4S] cluster at *g* = 1.97) increase in intensity, indicating the illumination is capable of forcing the low-potential pathway to be loaded with electrons. Despite this overall increase, the first 10 minutes shows that the generation of the radical species precedes proximal [4Fe-4S] cluster reduction, which itself precedes distal [4Fe-4S] cluster reduction. Fitting parameters located in **Table S5** below

Table S5: Fitting parameters for the generation of the paramagnetic species in the first 10 minutes of the phototitration experiment shown above in **Figure S16**. Fits were performed using **Equation S8** below.

Fitting Parameter	Radical*	Proximal Cluster	Distal Cluster
y_0	0.245 ± 0.004	0.161 ± 0.003	0.29 ± 0.02
A (intensity)	-0.242± 0.005	-0.159± 0.004	-0.29 ± 0.02
τ (min)	0.85 ± 0.04	2.0 ± 0.1	3.2 ± 0.6
χ^2	$0.176 \bullet 10^{-4}$	$0.131 \bullet 10^{-4}$	$2.36 \bullet 10^{-4}$
R^2	0.99879	0.99775	0.98682

*Radical fit based on 100 K data, whereas proximal and distal clusters were based on 20 K data.

$$\boldsymbol{Normalized\ \Delta Intensity}\ (\%) = \boldsymbol{y_0} + \boldsymbol{A} * \boldsymbol{e}^{\left(-\frac{(x)}{\tau}\right)} \quad \textbf{Equation S8}$$

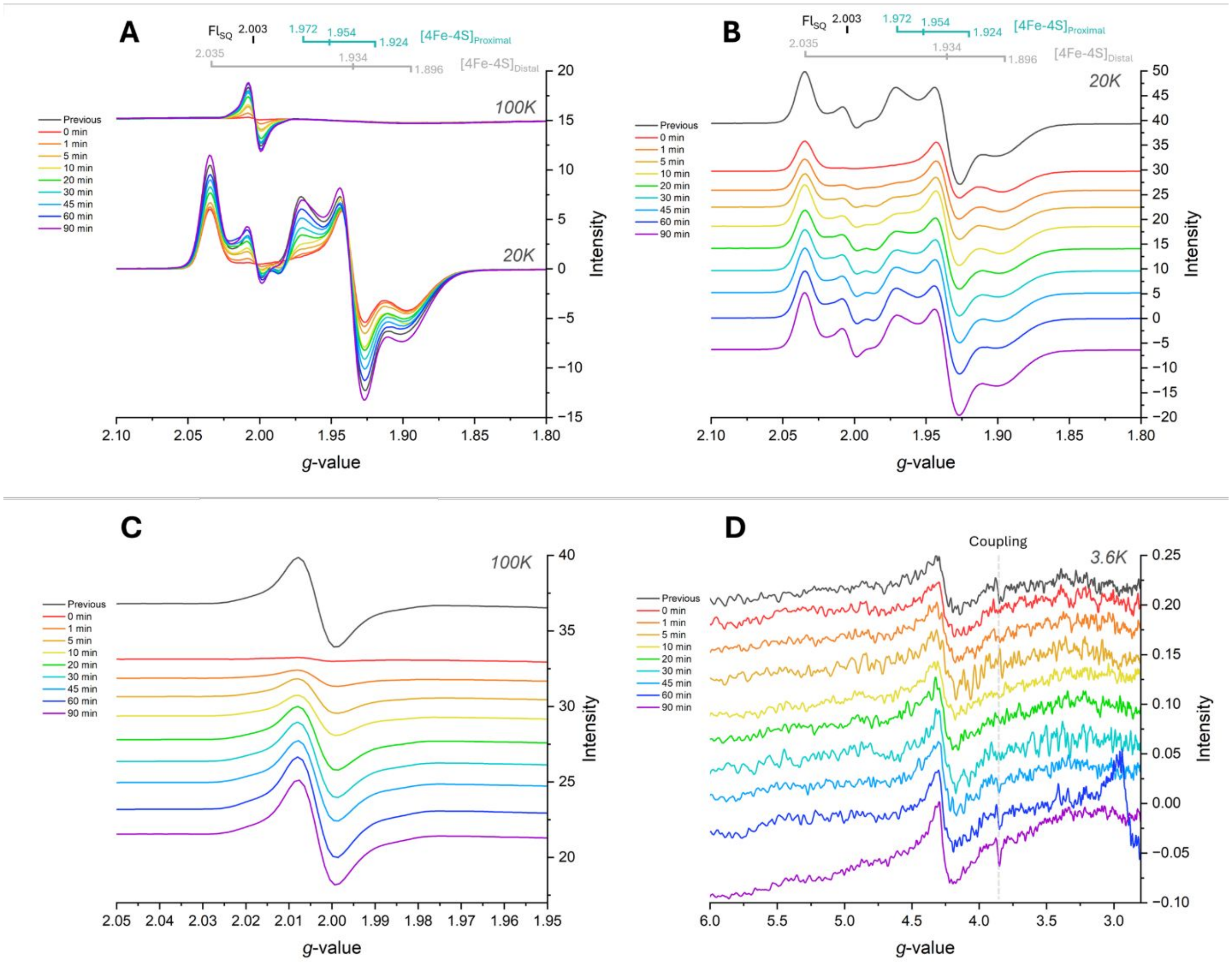

<u>Figure S17:</u> Second phototitration experiment performed on the same sample used in Figure 4. Storage spectra (*grey*) are the same conditions as the 90-minute illumination seen in **Figure 4**, but with the addition of 24 days of liquid nitrogen storage at ~ 77 K. Panels **A-D** show the general replicability of the illumination experiment after annealing at RT, reproducing the data observed in **Figure 4** closely. Panel **D** depicts another anomalous spectral feature at *g* ~ 2.9 for the 60-minute trace that disappears before the next trace, as an artifact of unknown origin. Panel **D** also validates the anomalous feature at *g* ~ 3.7 in **Figure 4D** is likely an irrelevant spectral artifact, since the feature in question is absent from this phototitration's 90-minute spectrum.

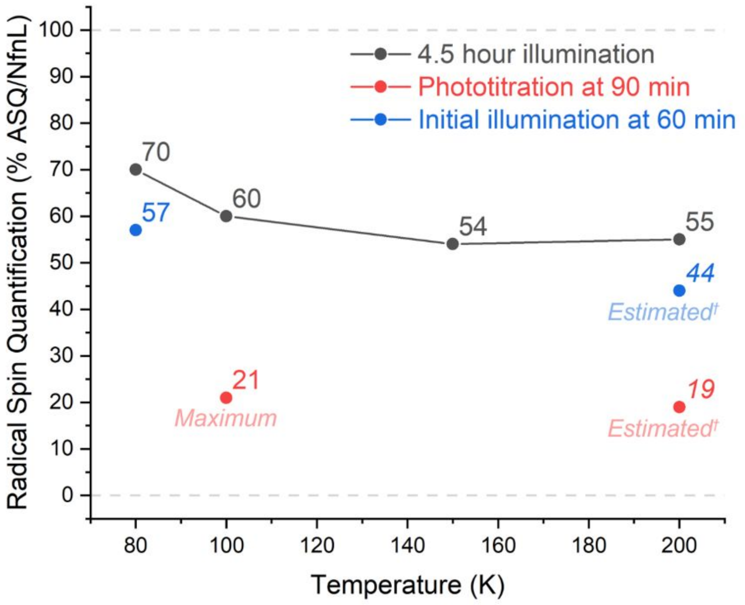

<u>Figure S18:</u> Low-temperature photoexcitation generates significant populations of an ASQ on the L-FAD. All ASQ radical spins per NfnL were quantified relative to a Cu(II) TEA standard at a concentration of 100 µM at the exact spectral acquisition parameters used for the radical sample. Due to higher radical signal saturation at lower temperatures, the total spin percentage of the ASQ generally decreases as the temperature increases. Assuming the highest temperature quantified (200K) is the most accurate to the true amount of ASQ per NfnL, the value is quantified to 55% for the 4.5 hour illumination condition. †Estimated spin quantities at 200 K for the phototitration (19%) and the initial illumination (44%) were extrapolated by using the temperature relationship for the 4.5 hour illumination sample, where the “X” temperature was 80 K for the initial photoillumination and 100 K for the phototitration at 90 min (**Equation S9**).

$$\boldsymbol{Est.\,Spin\,Quant._{200K}}\ (\%) = \left(\frac{\%\ \boldsymbol{of 4.5\ illum_{200K}}}{\%\ \boldsymbol{of\ 4.5\ illum_{"X"K}}}\right) * \%\ \boldsymbol{of\ sample_{"X"K}} \qquad \textbf{Equation S9}$$

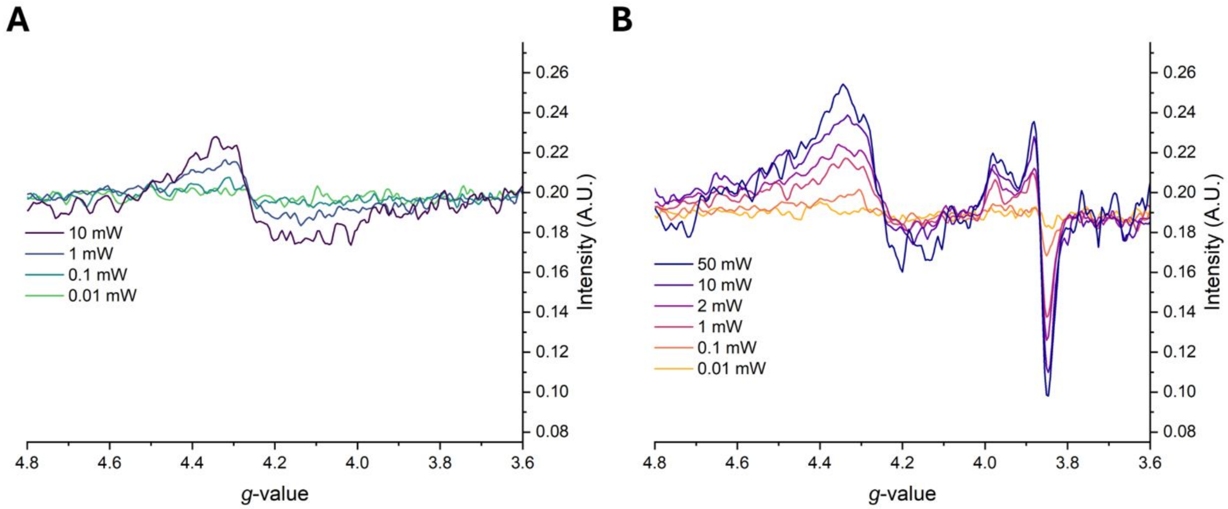

<u>Figure S19:</u> Low field EPR data at 3.6 K for 1x DTH (*left*, **A**) and 10x DTH (*right*, **B**) corresponding to **Figure S1A** and **Figure S3A**. As mentioned in **Figure 6**, the cluster-cluster coupling feature is only observed when both proximal and distal [4Fe-4S] clusters are reduced and observable in the *g* ~ 2 region.[17] Signal present at *g* ~ 4.3 is from adventitiously-bound Fe^{3+} (e.g., "junk iron") and is unrelated to any enzymatic or mechanistically relevant signals.

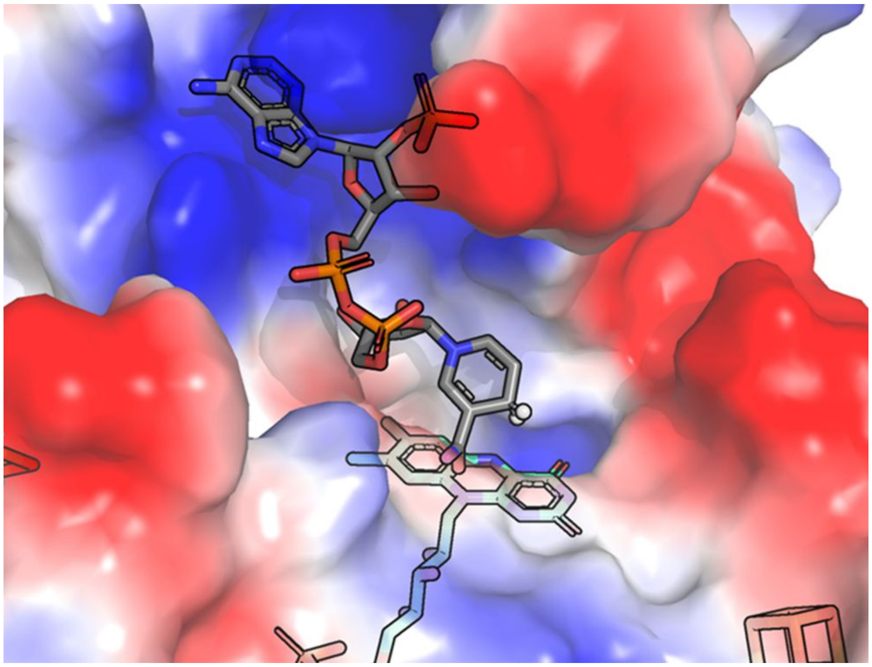

Figure S20: Crystal structure of NADP(H)-bound NfnSL, highlighting the proximity of the pyridine nucleotide substrate to the L-FAD in NfnL.[7] The NADP(H) has been modeled as NADPH with the donatable hydrides shown as grey spheres for illustrative purposes to show the transferable hydride is very close to the N5 position on the L-FAD. This proximity of the NADPH hydride donor provides context for how the photoexcitation mechanism is proposed to facilitate unstable reduced intermediates observed in this report. The photoexcited L-FAD is proposed to accept electrons from NADPH which then transfers the electron to the proximal [4Fe-4S] cluster, which then the NADP$^{\bullet}$ re-reduces the L-FAD to an ASQ. PDB: 5JCA.

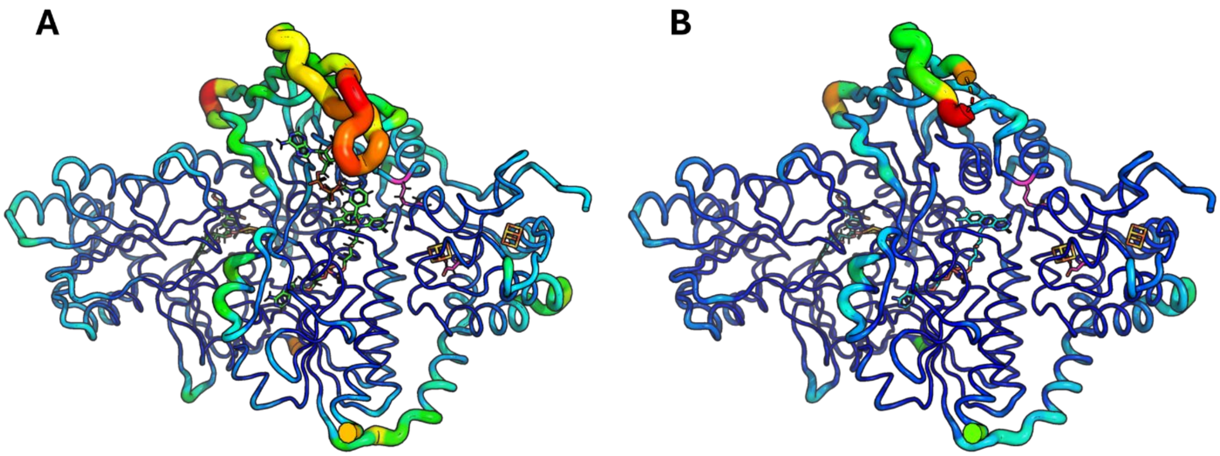

Figure S21: NADPH binding leads to increased structural movement in NfnSL. Panel **A** shows the crystal structure of NADPH-bound NfnSL, and panel **B** shows NfnSL without any substrates bound.[7] The presence of NADPH leads to the increase in β-factor (redder hues) in the loop corresponding to the ~300 to 400 region relative to that of the unbound NfnSL. R333 and E126 are highlighted pink in both structures. NADPH bound NfnSL is PDB: 5JCA; Unbound NfnSL is PDB: 5JFC.

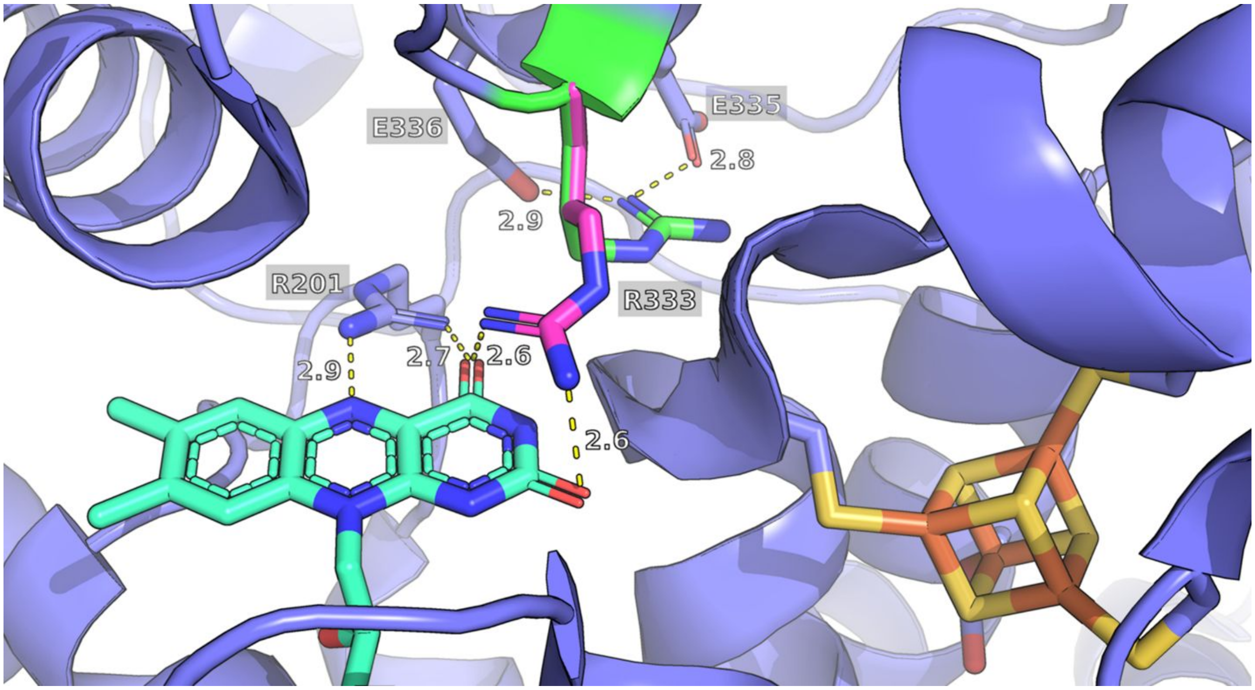

<u>Figure S22:</u> Arginine 333 (R333) can rotate and further electrostatically stabilize the L-FAD. R333 normally faces away from the L-FAD,[7] interacting with two glutamic acid residues E335 and E336 (*green*). The arginine's guanidine group may rotate over the flavin, poising itself 2.6 Å away from each of the O2' and O4' positions of the L-FAD (*magenta*), possibly further stabilizing high energy intermediates present on the L-FAD. PDB: 5JFC.

Supporting References:

(1) Elliott, J. I.; Brewer, J. M. The Inactivation of Yeast Enolase by 2,3-Butanedione. *Arch. Biochem. Biophys.* **1978**, *190* (1), 351–357. https://doi.org/10.1016/0003-9861(78)90285-0.

(2) Wiley, S.; Griffith, C.; Eckert, P.; Mueller, A. P.; Nogle, R.; Simpson, S. D.; Köpke, M.; Can, M.; Sarangi, R.; Kubarych, K.; Ragsdale, S. W. An Alcove at the Acetyl-CoA Synthase Nickel Active Site Is Required for Productive Substrate CO Binding and Anaerobic Carbon Fixation. *J. Biol. Chem.* **2024**, *300* (8). https://doi.org/10.1016/j.jbc.2024.107503.

(3) Wiley, S. A.; Spackman, I. J.; Lubner, C. E. Differential Ligation Alters Electronic State and Coupling Signals of Iron-Sulfur Clusters in Flavin-Based Electron Bifurcation. *J. Inorg. Biochem.* **2026**, *274*, 113051. https://doi.org/10.1016/j.jinorgbio.2025.113051.

(4) Galli, C.; Innes, J. B.; Hirsh, D. J.; Brudvig, G. W. Effects of Dipole–Dipole Interactions on Microwave Progressive Power Saturation of Radicals in Proteins. *J. Magn. Reson. B* **1996**, *110* (3), 284–287. https://doi.org/10.1006/jmrb.1996.0044.

(5) Brudvig, G. W. [22] Electron Paramagnetic Resonance Spectroscopy. In *Methods in Enzymology*; Academic Press, 1995; Vol. 246, pp 536–554. https://doi.org/10.1016/0076-6879(95)46024-1.

(6) Rupp, H.; Rao, K. K.; Hall, D. O.; Cammack, R. Electron Spin Relaxation of Iron-Sulphur Proteins Studied by Microwave Power Saturation. *Biochim. Biophys. Acta BBA - Protein Struct.* **1978**, *537* (2), 255–269. https://doi.org/10.1016/0005-2795(78)90509-3.

(7) Lubner, C. E.; Jennings, D. P.; Mulder, D. W.; Schut, G. J.; Zadvornyy, O. A.; Hoben, J. P.; Tokmina-Lukaszewska, M.; Berry, L.; Nguyen, D. M.; Lipscomb, G. L.; Bothner, B.; Jones, A. K.; Miller, A.-F.; King, P. W.; Adams, M. W. W.; Peters, J. W. Mechanistic Insights into Energy Conservation by Flavin-Based Electron Bifurcation. *Nat. Chem. Biol.* **2017**, *13* (6), 655–659. https://doi.org/10.1038/nchembio.2348.

(8) Wise, C. E.; Ledinina, A. E.; Mulder, D. W.; Chou, K. J.; Peters, J. W.; King, P. W.; Lubner, C. E. An Uncharacteristically Low-Potential Flavin Governs the Energy Landscape of Electron Bifurcation. *Proc. Natl. Acad. Sci.* **2022**, *119* (12), e2117882119. https://doi.org/doi:10.1073/pnas.2117882119.

(9) Buckel, W.; Thauer, R. K. Energy Conservation via Electron Bifurcating Ferredoxin Reduction and Proton/Na+ Translocating Ferredoxin Oxidation. *Evol. Asp. Bioenerg. Syst.* **2013**, *1827* (2), 94–113. https://doi.org/10.1016/j.bbabio.2012.07.002.

(10) Fournel, A.; Gambarelli, S.; Guigliarelli, B.; More, C.; Asso, M.; Chouteau, G.; Hille, R.; Bertrand, P. Magnetic Interactions between a [4Fe–4S]1+ Cluster and a Flavin Mononucleotide Radical in the Enzyme Trimethylamine Dehydrogenase: A High-Field Electron Paramagnetic Resonance Study. *J. Chem. Phys.* **1998**, *109* (24), 10905–10913. https://doi.org/10.1063/1.477786.

(11) Salerno, J. C.; Ohnishi, T.; Lim, J.; Widger, W. R.; King, T. E. Spin Coupling between Electron Carriers in the Dehydrogenase Segments of the Respiratory Chain. *Biochem. Biophys. Res. Commun.* **1977**, *75* (3), 618–624. https://doi.org/10.1016/0006-291X(77)91517-0.

(12) Duggan, D. Michael.; Hendrickson, D. N. Magnetic Exchange Interactions in Transition Metal Dimers. III. Nickel(II) Di-.Mu.-Cyanato, Di-.Mu.-Thiocyanato, and Di-.Mu.-Selenocyanato Complexes and Related Outer-Sphere Copper(II) Complexes. *Inorg. Chem.* **1974**, *13* (12), 2929–2940. https://doi.org/10.1021/ic50142a031.

(13) Wise, C. E.; Ledinina, A. E.; Lubner, C. E. Site-Differentiated Iron-Sulfur Cluster Ligation Affects Flavin-Based Electron Bifurcation Activity. *Metabolites* **2022**, *12* (9), 823.

(14) Grady, J. K.; Chasteen, N. D.; Harris, D. C. Radicals from "Good's" Buffers. *Anal. Biochem.* **1988**, *173* (1), 111–115. https://doi.org/10.1016/0003-2697(88)90167-4.

(15) Hausladen, D. M.; Peña, J. Organic Buffers Act as Reductants of Abiotic and Biogenic Manganese Oxides. *Sci. Rep.* **2023**, *13* (1), 6498. https://doi.org/10.1038/s41598-023-32691-5.

(16) Kirsch, M.; Lomonosova, E. E.; Korth, H.-G.; Sustmann, R.; de Groot, H. Hydrogen Peroxide Formation by Reaction of Peroxynitrite with HEPES and Related Tertiary Amines: IMPLICATIONS FOR A GENERAL MECHANISM*. *J. Biol. Chem.* **1998**, *273* (21), 12716–12724. https://doi.org/10.1074/jbc.273.21.12716.

(17) Mathews, R.; Charlton, S.; Sands, R. H.; Palmer, G. On the Nature of the Spin Coupling between the Iron-Sulfur Clusters in the Eight-Iron Ferredoxins. *J. Biol. Chem.* **1974**, *249* (13), 4326–4328. https://doi.org/10.1016/S0021-9258(19)42521-0.
